# Within-host competition causes pathogen molecular evolution and perpetual microbiota dysbiosis

**DOI:** 10.1093/ismejo/wraf071

**Published:** 2025-04-17

**Authors:** Emily J Stevens, Jingdi D Li, Tobias E Hector, Georgia C Drew, Kim Hoang, Samuel T E Greenrod, Steve Paterson, Kayla C King

**Affiliations:** Department of Biology, University of Oxford, Oxford, Oxfordshire, OX1 3SZ, United Kingdom; School of Life Sciences, Keele University, Keele, Staffordshire, ST5 5BG, United Kingdom; Department of Biology, University of Oxford, Oxford, Oxfordshire, OX1 3SZ, United Kingdom; Department of Biology, University of Oxford, Oxford, Oxfordshire, OX1 3SZ, United Kingdom; Department of Biology, University of Oxford, Oxford, Oxfordshire, OX1 3SZ, United Kingdom; Division of Infectious Diseases, Emory University School of Medicine, Atlanta, GA, 30322, United States; Department of Biology, University of Oxford, Oxford, Oxfordshire, OX1 3SZ, United Kingdom; Institute of Infection, Veterinary, and Ecological Sciences, University of Liverpool, Liverpool, Wirral, CH64 7TE, United Kingdom; Department of Biology, University of Oxford, Oxford, Oxfordshire, OX1 3SZ, United Kingdom; Department of Zoology, University of British Columbia, Vancouver, BC, V6T 1Z4, Canada; Department of Microbiology and Immunology, University of British Columbia, Vancouver, BC, V6T 1Z3, Canada

**Keywords:** competition, experimental evolution, pathogenesis, microbiota, host-pathogen interactions, molecular evolution, virulence

## Abstract

Pathogens newly invading a host must compete with resident microbiota. This within-host microbial warfare could lead to more severe disease outcomes or constrain the evolution of virulence. By passaging a widespread pathogen (*Staphylococcus aureus*) and a natural microbiota community across populations of nematode hosts, we show that the pathogen displaced microbiota and reduced species richness, but maintained its virulence across generations. Conversely, pathogen populations and microbiota passaged in isolation caused more host harm relative to their respective no-host controls. For the evolved pathogens, this increase in virulence was partly mediated by enhanced biofilm formation and expression of the global virulence regulator *agr*. Whole genome sequencing revealed shifts in the mode of selection from directional (on pathogens evolving in isolation) to fluctuating (on pathogens evolving in host microbiota). This approach also revealed that competitive interactions with the microbiota drove early pathogen genomic diversification. Metagenome sequencing of the passaged microbiota shows that evolution in pathogen-infected hosts caused a significant reduction in community stability (dysbiosis), along with restrictions on the co-existence of some species based on nutrient competition. Our study reveals how microbial competition during novel infection could determine the patterns and processes of evolution with major consequences for host health.

## Introduction

During host invasion, pathogens encounter a microbial community (i.e. microbiota) occupying the intended niche. Unlike resident microbiota, novel pathogens are not locally adapted. This lack of evolutionary history with the host environment may put these pathogens at a competitive disadvantage. Indeed, competitive exclusion of pathogens, and thus colonisation resistance by microbiota is a common phenomenon observed across a range of animal hosts [1, 2]. To overcome this ecological challenge, pathogens have been shown to kill competitors via toxins (i.e., interference competition) or provoke host inflammation in a type of “proactive invasion” (reviewed in [3, 4]). Pathogens can also compete with microbiota for host resources [5, 6]. These strategies might push out resident microbiota, reducing their numbers and diversity [4, 7], and contribute to high pathogen virulence during acute infection [8, 9]. The potential for competitive interactions between emerging pathogens and host microbiota to affect disease severity is unclear, but has generated considerable interest for managing the harm caused by infection in human medicine [10, 11], wildlife conservation [12], and agriculture [13]. Within-host microbial warfare can thus come with huge fitness implications for hosts, microbiota, and pathogens [14].

The evolutionary outcomes of microbiota-pathogen competition on disease severity are complex. Higher pathogen virulence might evolve over time in a more competitively exclusive microbiome [14], or be limited if already at a high optimum [15]. However, pathogens should be favoured to exploit their hosts cautiously to avoid killing them prematurely [16]. Microbiota of healthy hosts can exhibit rapid evolutionary dynamics in response to aging and diet [17], with resource competition as a strong source of selection [18, 19]. In the presence of competition with pathogens, individual microbial commensal species can become more competitive [20] and/or protective [21]. Alternatively, resource competition might limit microbial evolutionary responses [22, 23]. Within-host evolutionary interactions between pathogens and microbiota may be an important process underlying the transition from commensalism to pathogenicity [24].

Here, we directly tested whether pathogen competition with host microbiota could shape the evolution of disease severity. We experimentally evolved a widespread, disease-causing animal pathogen (*Staphylococcus aureus*) using a *Caenorhabditis elegans* nematode worm model of infection. *C. elegans* are likely exposed to *Staphylococcus* spp. in natural environments [25, 26]. However, in our model, *S. aureus* acts as a novel invading pathogen to canonical laboratory *C. elegans* nematodes. Pathogenic *S. aureus* strains are known to infect a diversity of animal host species, including domestic and wild animals [26–28], where they can engage in resource (or in some cases toxin-mediated) competition with other microbes [29, 30]. Nematodes infected by *S. aureus* are harmed when the pathogen accumulates and produces toxins in the host intestine [31, 32]. To independently test whether microbiota could become more exclusionary during *S. aureus* infection, we also passaged a constructed community of microbes within host populations [33]. This community consisted of seven bacterial species representing some of the most commonly found wild, resident *C. elegans* gut components (CeMBio collection [33]). Across short and longer-term time scales, we examined the eco-evolutionary trajectories of each passaged pathogen population and microbiota community in the context of within-host competition and virulence. The molecular and mechanistic basis of microbial adaptive processes were also explored.

## Materials and methods

### Species and strains used

*C. elegans* is an established model host for microbial colonisation and pathogenesis [34, 35]. The homogenous N2 line was maintained as standard and fed *Escherichia coli* strain OP50. This animal was evolutionarily static in this experiment and freshly resurrected at each passage.

These animals were exposed to a microbiota community originating from the CeMBio collection [33]. Seven species from this community were included in our experiment: *Pantoea nemavictus* (BIGb393), *Lelliottia amnigena* (JUb66), *Sphingobacterium multivorum* (BIGb0170), *Enterobacter hormaechei* (CEent1), *Acinetobacter guillouiae* (MYb10), *Pseudomonas lurida* (MYb11), and *Ochrobactrum pecoris* (MYb71). Worms exposed to microbiota were not fed OP50. Each microbiota component was selected based on their ability to grow on Xylose Lysine Deoxycholate (XLD) media to distinguish them from the pathogen, *S. aureus*.

Strain MSSA476 (GenBank: BX571857.1) was used as the pathogen, sourced from the University of Liverpool. Previous work has shown that *S. aureus* is an effective pathogen of *C. elegans* [20, 34]. Accumulation in the nematode gut has been correlated with increased host mortality [34], causing enterocyte effacement, intestinal epithelium destruction, and degradation of internal organs [31]. Expression of the quorum-sensing global virulence regulatory system *agr* has been implicated as a requirement for full pathogenicity in this nematode model [32, 36].

### Worm and bacterial growth conditions

Unless specified otherwise, all microbiota strains and OP50 were cultured in Luria-Bertani (LB) broth, inoculated with a single colony and incubated at 25°C (shaking 150 rpm). *S. aureus* was cultured in Todd Hewitt broth (THB) from a single colony and incubated at 30°C (shaking 150 rpm). On agar plates, microbiota and OP50 were cultured at 25°C on LB agar to obtain single colonies, whilst *S. aureus* was cultured at 30°C on Tryptic Soy Agar (TSA). Evolved bacterial populations used in follow-up host mortality assays were inoculated into liquid media directly from the −80°C archive. All other incubation conditions remained the same.

To isolate and sterilize eggs, gravid *C. elegans* worms were suspended in 6 ml M9 buffer containing 0.1% Triton-X (M9-Tx) and treated with 1 ml bleach (1:1 mix of 5 M sodium hydroxide and sodium hypochlorite). Worms were incubated in bleach at room temperature for up to 10 minutes, centrifuged (2 mins, 400 × g), and washed twice with M9-Tx. Worms were then resuspended in plain M9 and incubated at 20°C overnight (shaking 150 rpm). This allowed eggs to hatch and synchronized the population, such that all worms were arrested at the L1 larval stage. Hatched worms were transferred onto NGM plates seeded with 600 μl OP50 and incubated at 20°C for 48 h. L4 worms were then transferred to infection plates as described below.

### Evolution experiment design

**Figure 1:**
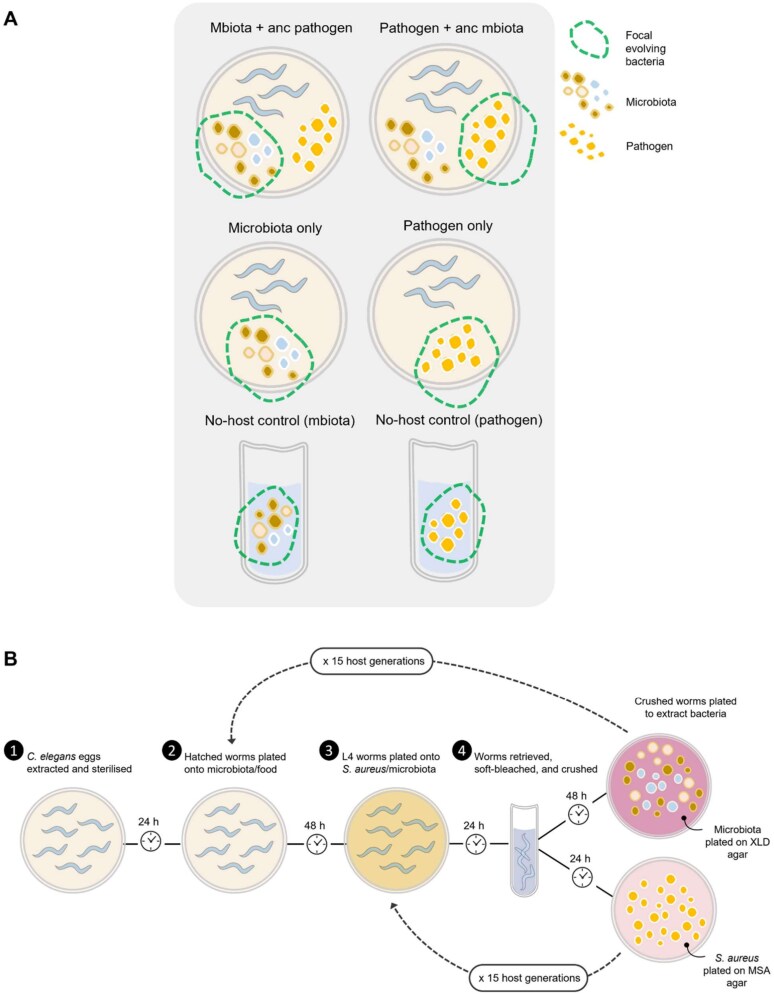
(**A**) Overview of each evolving group in the experiment. (**B**) Overview of the experimental evolution method.

The evolution experiment consisted of six different groups of passaged bacteria; four in vivo treatments and two no-host controls, each replicated six times (Fig. 1A). Briefly, both pathogen and microbiota were evolved alongside their ancestral opponent (“Pathogen + anc mbiota” and “Mbiota + anc pathogen”) and in isolation (“Pathogen only” and “Mbiota only”). No-host controls consisted of the pathogen and microbiota each evolving in vitro in isolation. In vivo evolving lines were passaged 15 times through non-evolving *C. elegans* populations (Fig. 1B). No-host controls were passaged alongside, acting as proxies for the ancestor(s) and controlling for lab adaptation.

Ancestral bacteria were re-introduced at each passage where relevant. For groups including a microbiota, each microbiota species was cultured individually, then standardized to the optical density (OD600) of the species with the lowest growth and pooled into one tube. For the Pathogen only group the food source was *E. coli* OP50, also standardized in line with the microbiota, to ensure no disparity in the amounts of bacteria available as food. 600 μl of food (pooled microbiota or OP50) was plated onto 9 cm NGM plates. Approximately 1000 hatched L1 worms were added to each plate, except the No-host control group, and all plates were incubated at 20°C for 48 h. A further 100 μl of the pooled microbiota community was spread onto additional NGM plates for use as substitute infection plates for the Mbiota only group. This ensured worms were fed the same microbiota community throughout the cycle (these plates were incubated for 24 h at 20°C then at 4°C until used at the infection step). To create infection plates for groups including the pathogen, *S. aureus* cultures were made and, following incubation, 100 μl was spread onto 9 cm TSA plates. These plates were incubated for 24 h at 30°C.

After 48 h incubation on food, worms were washed off plates with 850 μl M9-Tx. Worms were then transferred to cut-off 1000 μl filter tips within 1.5 ml tubes and washed twice with 250 μl M9-Tx (centrifugations were 2 mins, 290 × g). They were resuspended in 100 μl M9-Tx and transferred to either infection plates or to microbiota plates. All were incubated at 25°C for 24 h.

A gentle bleaching protocol was conducted as described in^33^ to remove any surface-adherent bacteria from the worm cuticle. This procedure ensured that, as far as possible, only gut bacteria were passaged. Worms were transferred in 100 μl M9 buffer (containing 0.025% Tx) and crushed using a Bead Bug Microtube Homogeniser (Benchmark Scientific) for 3 mins at 320 rpm. Approximately 100 worms remained in each replicate, representing 10% of the original population. Ten-fold serial dilutions were made of the crushed worm solution. 10^−2^ dilutions were plated onto XLD selective media to isolate the microbiota and 10^−4^ dilutions were plated onto MSA to isolate the pathogen. For the no-host controls, an inoculation loop was used to transfer a sample from the bacterial lawn onto XLD for microbiota and MSA for pathogen exposures. XLD plates were incubated for 48 h at 25°C, whereas MSA plates were incubated at 30°C for 24 h.

To passage bacteria to the next host generation, 100 colonies were picked from selective plates for microbiota and pathogen, for inoculation into 10 ml LB or THB, respectively. For no-host controls, an inoculation loop was used to take a sample from the bacterial lawn, which was inoculated into 10 ml liquid media. Aliquots of these liquid cultures were archived in 25% glycerol at −80°C. Liquid cultures were then used to make the next passage’s food/infection plates as described above. OP50 and evolving microbiota cultures were standardized to the ancestral species with the lowest growth from passage 2 onwards, to ensure the same amount of food was plated for all lineages.

### Host mortality assays

Pathogen virulence in the presence and absence of microbiota was evaluated by calculating the proportion of dead worms in a population exposed to *S. aureus*. Infection/microbiota plates were made as described above. However, 60 μl *S. aureus* was plated onto smaller 6 cm TSA plates and, for the evolved microbiota, only 400 μl liquid culture was plated onto NGM. After 24 h incubation the number of live and dead worms in a random half of each infection plate were counted. Raw data were then doubled. Three to six technical replicates were conducted for each sample.

### Bacterial growth assays

To assay bacterial colonisation of the worm gut, the evolution experiment protocol was followed up to the gentle bleaching step. Five worms were then sampled from each replicate and transferred to 100 μl M9. Worms were crushed (as per the protocol described above) and serial dilutions prepared. For each sample at each dilution, three 10 μl spots were plated onto MSA/XLD agar to count the number of colony forming units (CFU per worm).

Growth of the microbiota and pathogen, both individually and in competition, was assayed in vitro. Each species was cultured separately overnight, standardized to the same optical density (OD600), and pooled as described above. Of the pooled microbiota and/or pathogen, 1.5 μl was added to 1.5 ml LB broth. These cultures were incubated for 24 h at 25°C to replicate the infection conditions of the in vivo experiments. Cultures were then serially diluted 10-fold and spots plated as described above.

### Pathogen biofilm assay

Biofilm formation of ancestral and evolved *S. aureus* was assessed. Evolved pathogens from passage 15 were inoculated directly from freezer stocks into 10 ml THB broth and incubated at 30°C with shaking for 24 h. Cultures were then diluted 1:40 into 100 μl THB containing 0.5% glucose in a 96-well plate. Wells at the edges of the plate were filled with sterile water to prevent evaporation of the samples and the plate was incubated statically for 24 h at 30°C. Plates were incubated inside a plastic box lined with wet paper towel. Positive (*S. aureus* MSSA476) and negative (*S. aureus* LAC) controls were included. After incubation, samples were washed once with sterile water, stained with 150 μl 0.5% crystal violet for 30 mins at room temperature, washed again, and resuspended in 200 μl 7% acetic acid. OD(595) readings were taken for each sample.

### Metagenomic sequencing of evolved microbiota

Metagenome sequencing of microbiota was conducted on passage 15 for the microbiota communities. 10 ml LB cultures were inoculated straight from the evolved microbiota freezer stocks. Cultures were incubated overnight at 25°C, with shaking. Cultures were then pelleted by centrifugation, resuspended in 1 ml PBS, and incubated with 25 units mutanolysin overnight at 37°C, with shaking. Samples were then digested with 0.5 μg RNase A for 15 minutes at room temperature. DNA was extracted using the High Pure PCR Template Preparation Kit (Roche) as per the manufacturers protocol.

Sequencing was conducted by the Centre for Genomic Research at the University of Liverpool, UK. Extracted DNA was prepared for short-read sequencing using the NEBNext Ultra II Kit, using 1/2 volume reactions. DNA was sequenced on a NovaSeq System (Illumina) using S4 chemistry (Paired-end, 2x150 bp sequencing, generating an estimated 2000 million clusters per lane). Raw FASTq files were trimmed for the presence of Illumina adapter sequences using Cutadapt version 1.2.1 [37]. The option -O 3 was used, so the 3′ end of any reads which match the adapter sequence for 3 bp. or more are trimmed. The reads were further trimmed using Sickle version 1.200 with a minimum window quality score of 20. Reads shorter than 15 bp. after trimming were removed.

Reference sequences were downloaded for each bacterial species. Trimmed reads were mapped to the reference genomes using bwa (identity> = 90% and coverage> = 60%). Reads mapped in proper pairs were extracted using samtools (−q 1 -f 2) [38]. The number of mapped reads were summed up for each bacterial species in each sample, using custom scripts. To calculate scaled relative abundance, raw count data were scaled by reference genome length, to account for the difference in genome length for different bacterial species. The scaled read count was then divided by the sum count for each sample. To determine the presence and absence of each bacterial strain, reads were mapped from samples of each ancestral species to the reference genomes. The cutoff was determined by the number of reads that were randomly mapped to different genomes.

### Pathogen whole-genome sequencing

Whole genome sequencing was conducted on evolved pathogen populations (40 pooled clones) from passages 10 and 15. Each clone was cultured separately and standardized to the same optical density before being pooled in equal volumes. Pooled populations were then pelleted and stored at −20°C until used for DNA extractions. Simultaneously, two individual clones were sequenced per population. Frozen pellets were resuspended in 200 μl PBS for DNA extraction. Extractions were conducted using the High Pure PCR Template Preparation Kit (Roche) as per the manufacturers protocol with two changes: 10 μg lysostaphin was added to lyse the cells for 30 mins instead of lysozyme and immediately following this samples were digested with 0.5 μg RNase A for 15 mins at room temperature.

Sequencing was conducted by the Centre for Genomic Research at the University of Liverpool. Extracted DNA was prepared for short-read sequencing using the NEBNext Ultra II FS Kit on the Mosquito platform, using a 1/10 reduced volume protocol. DNA was sequenced on a NovaSeq System using SP chemistry (Paired-end, 2 × 150 bp sequencing, generating an estimated 325 million clusters per lane). Raw FASTq files were trimmed for the presence of Illumina adapter sequences as described above.

Reads were further trimmed using FASTp [39]. Variant calling was conducted using the breseq pipeline (v. 0.36.0) [40]. Reads were mapped to the NCBI reference sequence NC_002953.3 (*S. aureus* subsp. *aureus* MSSA476). gdtools was used to generate a comparison table of the SNPs in each sample and a spreadsheet detailing the type and number of mutations in each sample.

Euclidean genetic distance, both between replicates and of each replicate from the ancestor, was calculated using the dist() function in R.

Negative frequency dependent selection was calculated by regressing the change in pathogen variant frequencies (%) from passages 10-15 with the observed frequency (%) at passage 10, following methods described previously [41, 42]. Grey shading around the regression line represents 95% confidence intervals.

### Pathogen qRT-PCR

Worms were colonized with either evolved or ancestral microbiota then infected with *S. aureus*, as described above. Four replicates were assayed from each group for this experiment—the four most virulent from the pathogen that evolved in isolation and the four least virulent from the no-host control. After 12 h incubation on infection/microbiota plates (time chosen for optimal gene expression), worms were washed off the plates with 3 ml M9-Tx and left to settle in a centrifuge tube. Worms were aspirated from tubes in 150 μl volumes and transferred to cut-off 1000 μl filter tips within 1.5 ml tubes. These tubes were centrifuged for 2 mins at 290 × g, worms were then resuspended in 600 μl RNA lysis buffer (Zymo) and transferred to ZR bead bashing tubes. Worms were lysed with a Disruptor Genie (full speed for 2 mins), then centrifuged for 5 mins at 16000 × g. RNA was extracted from lysed samples using the Zymo Quick RNA Miniprep kit, as per the manufacturers protocol, and stored at −80°C.

DNA was digested using the Turbo DNase digest kit, as per the manufacturers protocol. cDNA was synthesized from RNA using the AccuScript High-Fidelity First Strand cDNA Synthesis kit, as per the manufacturers protocol. 100 ng RNA was used for all cDNA synthesis reactions, to standardize the amount of RNA across samples. qPCR was conducted using the Luna qPCR Master Mix (NEB), with two technical replicates per cDNA sample. Briefly, 2 μl cDNA was added to a 20 μl reaction mix, containing 0.25 μM final concentration of each primer. RNAIII primers (FW: AGCATGTAAGCTATCGTAAACAAC and RV: TTCAATCTATTTTTGGGGATG) were used as the target gene and gyrB primers (FW: CAGCGTTAGATGTAGCAAGC and RV: CCGATTCCTGTACCAAATGC) were used as the housekeeping gene. Expression of RNAIII was calculated relative to the housekeeping gene using the 2^-∆∆CT^ method [43]. For the evolved pathogen qRT-PCR, one outlier was removed at the data analysis stage, leaving three replicates per group in total.

### Genome-scale metabolic modelling

To explore the potential nutritional overlap and metabolic interactions between each microbiota species and the pathogen, we reconstructed genome-scale metabolic models (GEMs) for each strain. For each strain, its annotated protein sequences were downloaded from NCBI, and were used for GEM reconstruction based on the top-down carving approach of curated “universal models” using CarveMe [44]. Pairwise potential cross-feeding interactions were evaluated using SMETANA scores, calculated using SMETANA (with 100 permutations). SMETANA scores can range from 0 (complete independence) to 1 (essentiality) for each metabolite, with higher scores indicating stronger unidirectional cross-feeding interactions from donor species to receiver species. For each bacterial pair, Metabolic Interaction Potential (MIP) and Metabolic Resource Overlap (MRO) were calculated using SMETANA. MIP calculates how many metabolites two species can share or exchange to decrease their dependency on external resources. MIP can be used to evaluate microbial cooperative metabolism. MIP and MRO estimate microbial interactions at their theoretical limit, regardless of the external environment.

### Statistics

All statistical analysis was conducted in R v4.2.2. Between 2-4 technical replicates were conducted for all assays and unless stated otherwise technical replicates were treated as independent data points. All data were tested for normality using the Shapiro–Wilk normality test. Normally distributed data was tested for statistical significance using parametric tests, including Welch Two-sample T-tests for datasets with two groups and ANOVA with post-hoc TukeyHSD tests for datasets with more than two groups. Non-normally distributed data was tested for statistical significance using non-parametric tests, including Wilcoxon rank sum tests for datasets with two groups and Kruskal–Wallis tests with post-hoc Dunn tests for datasets with more than two groups. Results were considered to be statistically significant when *P* < = 0.05. *P* values were corrected for multiple comparisons using the Benjamini-Hochberg method. When it was not relevant to compare all groups with each other, data was tested for statistical significance using a linear regression, and the “emmeans” package was used to specify groups for post-hoc analysis.

Plots were made using the “ggplot2” package in R [45]. Normally distributed data was plotted with the mean and standard error of the mean indicated on graphs. Non-normally distributed data was plotted with boxplots overlaying the datapoints to show the median and interquartile ranges. Whiskers of the boxplots represent 1.5 × interquartile range +/− third and first quartiles respectively. Points outside the whiskers are considered outliers.

## Results

### Presence of microbiota increases host mortality from *S. aureus* infection

**Figure 2:**
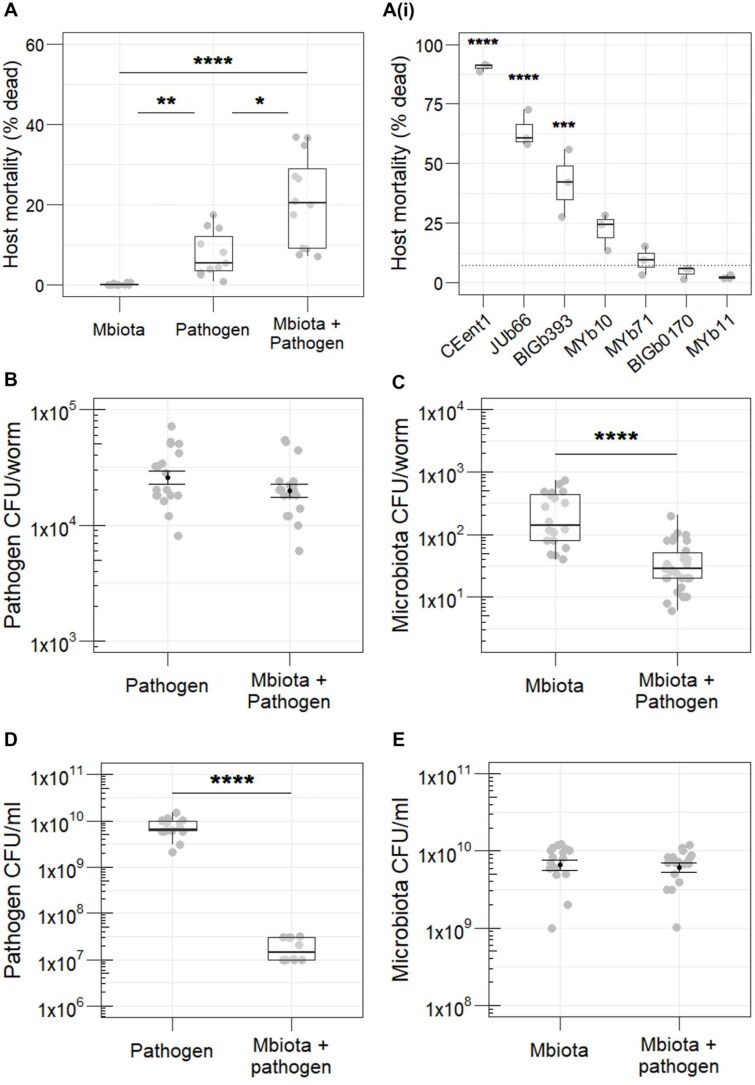
(**A**) Mortality of *C. elegans* from *S. aureus* is significantly higher in the presence vs absence of a 7-species microbiota community (n = 11-12, Kruskal–Wallis rank sum test, df = 2, *P* < 0.0001, Dunn test comparisons: Pathogen vs Mbiota + pathogen *P* = 0.053, pathogen vs Mbiota *P* = 0.003, Mbiota vs Mbiota + pathogen *P* < 0.0001). (**Ai**) Significant variation in the extent to which each microbiota component individually facilitates pathogen virulence in *C. elegans*. Significance is indicated only where species are significantly different to the control *E. coli* OP50, depicted by dotted line (n = 3, linear regression, *F* = 59.2, df = 16, *P* < 0.0001, pairwise comparisons using “emmeans” package: CEent1 vs control *P* < 0.0001, JUb66 vs control *P* < 0.0001, BIGb393 vs control *P* = 0.002). (**B**) In vivo colonisation of *C. elegans* by the pathogen is not significantly affected by the presence of the microbiota (n = 18, Welch two-sample T test *P* = 0.172), whereas (**C**) colonisation by the microbiota is significantly lower in the presence vs absence of the pathogen (n = 20-30, Wilcoxon rank sum test *P* < 0.0001), as measured by colony-forming units (CFU) per worm. (**D**) In vitro growth of the pathogen is significantly reduced in the presence vs absence of the microbiota in LB media (n = 12-18, Wilcoxon rank sum test *P* < 0.0001), as measured by CFU/ml. (**E**) In vitro growth of the microbiota is comparable in the presence and absence of the pathogen in LB media (n = 17-18, Welch two-sample T test *P* = 0.474).

We tested how disease severity from *S. aureus* was initially affected by host microbiota. When infected with *S. aureus,* we found mortality of *C. elegans* nematodes significantly increased in hosts pre-colonized by a microbiota community compared to *S. aureus* alone (Fig. 2A). Colonisation by the microbiota alone did not yield any host mortality (Fig. 2A). We determined whether this increased host-killing observed during infection was dependent on the whole microbiota community or one species. We exposed nematode populations to each microbiota species separately and infected them with *S. aureus*. We found considerable variation in the extent to which colonisation by each microbiota component contributed to host killing by the pathogen (Fig. 2Ai). Significantly higher host mortality was observed in nematodes colonized with CEent1 (*E. hormaechei*), JUb66 (*Lelliottia amnigena*), and BIGb393 (*Pantoea nemavictus*) during *S. aureus* infection, compared to the *E. coli* OP50 control (standard *C. elegans* food). Colonisation by strains MYb10 (*Acinetobacter gouilliae*), MYb71 (*O. pecoris*), BIGb0170 (*S. multivorum*), and MYb11 (*P. lurida*) did not result in significantly higher mortality during *S. aureus* infection. In the absence of *S. aureus*, these species do not individually cause mortality in *C. elegans* [33].

We tested whether differences in colonisation of *C. elegans* by the pathogen and microbiota determined disease severity. We extracted internal bacteria from surface-sterilized nematodes and counted the number of colony-forming units (CFU) per host. Pathogen load remained unchanged by the presence of host microbiota (Fig. 2B), indicating no relationship between pathogen-induced host mortality and in vivo load. The microbiota is a weaker competitor within the host, as we found total microbiota load was significantly reduced during pathogen infection (Fig. 2C). We observed the opposite dynamic during *in vitro* competition. Pathogen growth was suppressed in the presence of the microbiota (Fig. 2D), but growth of the microbiota was consistent regardless of *S. aureus* presence (Fig. 2E). The growth and competition dynamics between pathogen and microbiota are clearly dependent on the host environment.

### *S. aureus* becomes more virulent compared to controls when evolved in isolation

We hypothesized that competition with host microbiota would drive changes in pathogen virulence over evolutionary time. We passaged *S. aureus* either with a non-evolving (ancestral) 7-species microbiota or on its own, within genetically homogeneous, non-evolving *C. elegans* populations for 15 passages (Fig. 1). The 7-species microbiota community was similarly passaged with ancestral *S. aureus*. No-host controls were included to act as a proxy for the ancestor, controlling for any lab adaptation that may have occurred during the in vitro steps of the experiment. Pathogen populations started from the same clone of *S. aureus*, and microbiota communities from the same clone of each species. Evolution was thus dependent on *de novo* mutation and follow-on selection. At the end of each passage, 100 colonies of *S. aureus* and 100 colonies from the mixed microbiota communities were collected randomly from nematodes and used to start the next passage.

**Figure 3:**
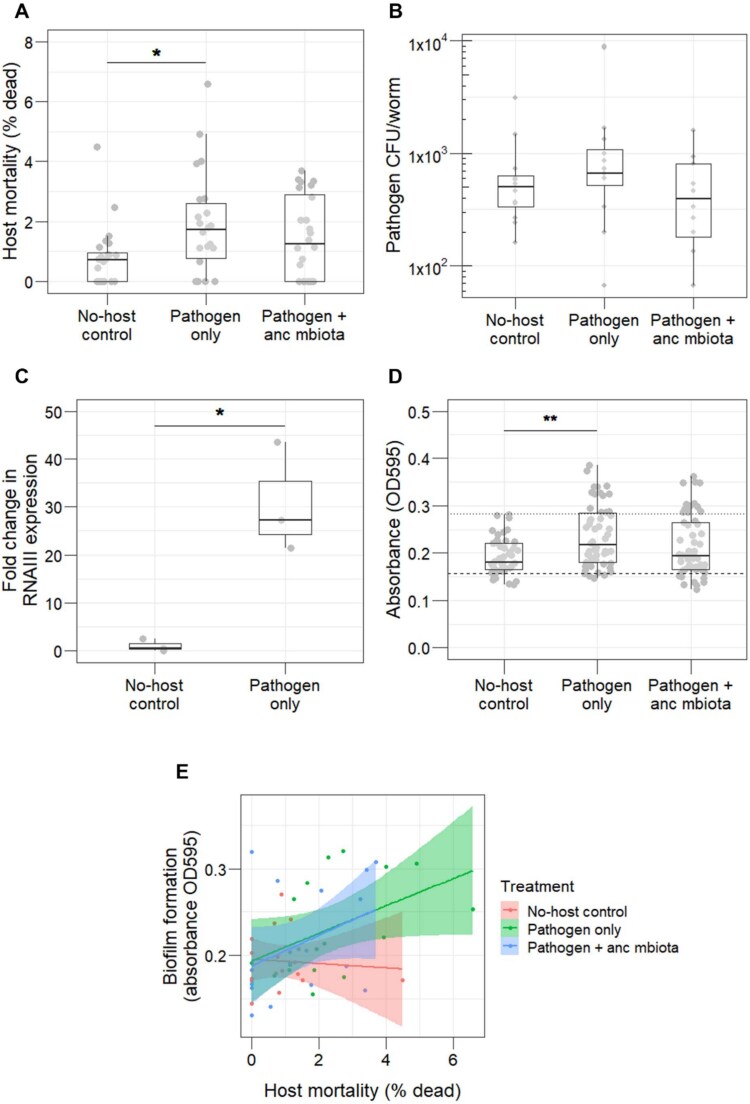
All data shown represents the 15th (final) evolved passage. (**A**) The pathogen only group was significantly more virulent in the absence of the microbiota than the No-host control (n = 22-24, Kruskal–Wallis rank sum test, df = 2, *P* = 0.038, Dunn test comparison No-host control vs pathogen only *P* = 0.038). (**B**) The difference in virulence is not mediated by bacterial colonisation of *C. elegans*, which is comparable across all evolved pathogens (n = 12-16, Kruskal–Wallis rank sum test, df = 2, *P* = 0.216). (**C**) Expression of the global virulence regulator *agr*, as measured by RNAIII expression, is significantly higher in the pathogen only group compared to the No-host control (n = 3, Welch two-sample T test *P* = 0.045). (**D**) Evolved pathogens were assayed for their ability to form biofilm in a 96-well plate, alongside the poor biofilm-forming strain LAC (negative control – Dashed line), and MSSA476 (positive control – Dotted line). The pathogen only group showed significantly more biofilm formation than the No-host control (n = 48-54, Kruskal–Wallis rank sum test, df = 2, *P* = 0.004), Dunn test comparison No-host control vs pathogen only *P* = 0.003). (**E**) Overall, there was a significant positive correlation (n = 16-18, Spearman’s rank correlation rho, *P* = 0.005) between biofilm formation and virulence phenotype of the evolved pathogens. When broken down by pathogen evolutionary history, the pathogen-only group showed a significant positive correlation (n = 17, Spearman’s rank correlation rho, *P* = 0.045) between these two phenotypes.

After 15 passages, we assayed each evolved pathogen for virulence (host mortality during infection) and colonisation ability. For all evolved pathogens, virulence was first measured in the absence of the ancestral microbiota. The pathogen evolved in isolation caused significantly higher mortality than the no-host control (Fig. 3A). In contrast, the pathogen evolved with ancestral microbiota caused similar levels of mortality to the no-host control (Fig. 3A). No significant difference in virulence was found between the pathogen that evolved in isolation compared to the pathogen that evolved with ancestral microbiota (Fig. 3A). All evolved pathogens exhibited a similar ability to colonize nematode hosts (Fig. 3B) suggesting pathogen accumulation did not determine harm caused.

### *Agr* activity and biofilm formation contribute to the increase in evolved *S. aureus* virulence

We found that in vivo RNAIII expression was on average 27-fold higher in the pathogen evolved alone compared to the no-host control (Fig. 3C). *agr* is a well-characterized global regulator of staphylococcal virulence [46, 47], and RNAIII is the effector of target gene regulation [48]. Activation of *agr* results in increased expression of secreted proteins involved in virulence [49]. We simultaneously tested RNAIII expression in the ancestral pathogen in the presence and absence of ancestral microbiota (Fig. S1a), to see if upregulation of *agr* explained the original increase in virulence (Fig. 2A). However, no difference was observed in *agr* expression in the pathogen that infected microbiota-colonized hosts (Fig. S1a).

We also assessed the ability to form in vitro biofilm of each of the evolved pathogens. Biofilm formation is a well-studied aspect of virulence in *S. aureus* [50, 51]. In nematodes, the formation of staphylococcal biofilm contributes to the establishment of infection via attachment to host cells and to resistance to host immune factors [52]. Thus, biofilm is an important contributor to virulence in this nematode model. This phenotype was measured using a 96-well plate assay, in which biofilms were stained with crystal violet after 24 h bacterial growth and quantified by optical density (595 nm). In comparison to the no-host control, the pathogen that evolved in isolation exhibited significantly higher biofilm formation (Fig. 3D), which positively correlated with its killing ability (Fig. 3E).

### No difference in virulence of evolved pathogens in hosts colonized by ancestral microbiota

When hosts were colonized by the ancestral microbiota, all evolved pathogens caused comparable levels of host mortality (Fig. S1b). All evolved pathogens also colonized worms better in the presence, compared to the absence, of ancestral microbiota (Fig. S1c). In contrast with the results described above, in which the ancestral pathogen load was equivalent in the presence and absence of microbiota (Fig. 2B), evolved pathogen load was consistently higher in the presence compared to the absence of microbiota (Fig. S1c). In keeping with the results described above (Fig. 2C), microbiota load was significantly reduced in infected compared to uninfected hosts (Fig. S1d). This result suggests that adaptation to the lab environment (not just host environment) favoured a competitive fitness advantage for *S. aureus*. We additionally characterized the effect of each evolved pathogen on microbiota community assembly. We found that differences in community assembly did not depend on the evolutionary history of the pathogen, but instead differed between *in vitro* and *in vivo* contexts (see Supplementary material: section 1).

### Genetic diversification of *S. aureus* is slower in the absence of microbiota

We next evaluated the molecular basis for differences in evolved pathogen virulence using whole genome sequencing. For all six replicates of each evolved pathogen, forty clones were pooled for population sequencing. Simultaneously, two clones were individually sequenced per replicate. Populations and clones were sequenced for passages 10 and 15 to allow characterisation of evolutionary changes over time. Variant calling was conducted from sequencing data to identify signatures of genomic evolution. We filtered out ancestral and no-host control SNPs from evolved lineages, to focus our analysis on *de novo* mutations.

**Figure 4:**
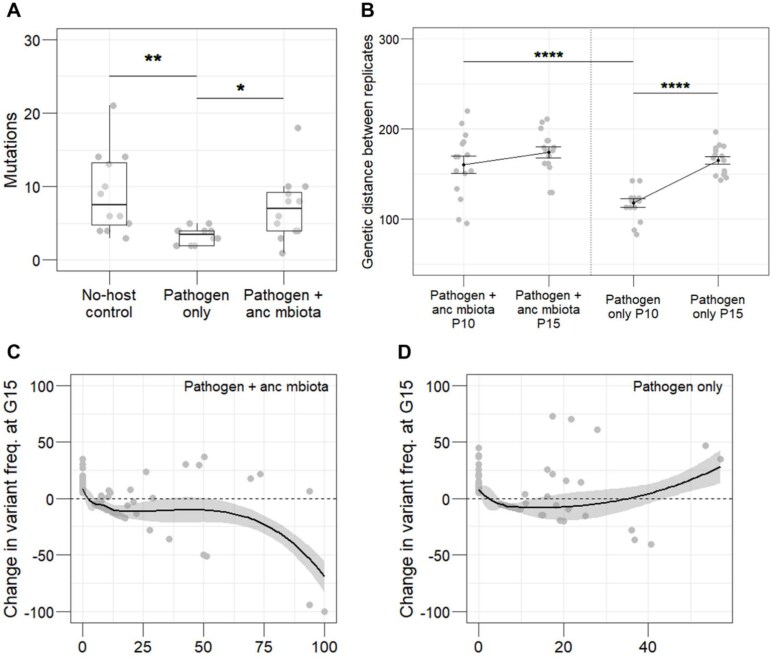
(**A**) After 15 passages, significantly fewer mutations occurred in clones of the pathogen only group compared to clones that evolved alongside the microbiota (n = 12, Kruskal–Wallis rank sum test, df = 2, *P* = 0.002, Dunn test comparisons: No-host control vs pathogen only *P* = 0.003, pathogen only vs pathogen + anc mbiota *P* = 0.017). (**B**) Genetic distance between replicate populations within each evolved group at generations 10 and 15. Significantly less genetic distance was found between replicates of the pathogen only group at generation 10 compared to generation 15 (n = 15, ANOVA, *F* = 15.3, df = 3, *P* < 0.0001, Tukey HSD comparison between pathogen only P10 vs P15 *P* < 0.0001) and compared to generation 10 of the pathogen + anc mbiota group (TukeyHSD *P* = 0.0001), indicating a slower rate of diversification in populations of the pathogen only group. (**C**) Negative frequency-dependent selection was observed when the pathogen evolved alongside the ancestral microbiota (n = 205, linear regression, *F* = 66.9, df = 1, *P* < 0.0001), but (**D**) not in the pathogen only group (n = 301, linear regression, *F* = 3.2, df = 1, *P* = 0.076).

Whole genome sequencing of clones at passage 15 revealed that significantly fewer mutations, specifically non-synonymous mutations and indels (Fig. S2a) occurred in pathogen clones evolved in isolation (Fig. 4A). These mutations were spread throughout the genome. Any SNPs present at > = 25% frequency within pooled populations of each replicate, after filtering of ancestral and no-host control SNPs, were classified as targets of selection (Table S1). We investigated which of these genes were connected to virulence in *S. aureus* (Supplementary Material: section 2). An analysis of nucleotide diversity was also conducted across experimental time points (Supplementary Material: section 3).

Molecular evolution in pathogens that evolved with ancestral microbiota initially led to rapid diversification. We calculated genetic distance among replicates for each of the in vivo evolved pathogens using population-level sequences. At passage 10, replicate populations in which the pathogen evolved with ancestral microbiota were more genetically distant from each other compared to replicates of the pathogen that evolved alone (Fig. 4B). Given that the evolution experiment started from a single ancestral clone, any alleles noted at high frequency at passage 10 would have increased from rare. By the final passage, the genetic distances among replicates were comparable between the two evolved pathogen groups (Fig. 4B). At both sampled time points, there was no significant difference in genetic distance to the ancestor between the two evolved pathogen groups (Fig. S2b). Taken together, these findings suggest that the presence of host microbiota can drive pathogen populations to evolve differently at the genomic level early on, with diversification persisting over a longer period of evolutionary time. Conversely, pathogen populations evolve more similarly without competition with host microbiota. Genomic differences across these populations accumulate and thus take longer to appear.

### Negative frequency-dependent selection acts on *S. aureus* in hosts with microbiota

We examined the modes of selection operating throughout the experiment, to further characterize pathogen evolutionary dynamics. For the pathogen that evolved with ancestral microbiota, approximately 30% of genes under selection fluctuated in frequency (higher frequency at passage 10 than 15) throughout the experiment (Fig. S2c). In contrast, only 12.5% of genes under selection fluctuated in the pathogen that evolved alone (Fig. S2d). Consequently, we looked for signatures of negative frequency-dependent selection using a standard approach [41, 42], in the pathogen that evolved in hosts with ancestral microbiota. Negative frequency-dependent selection (NFDS) occurs when rare variants are selected for within a population, thus individuals with the most common genotype are at a selective disadvantage [53, 54]. In a direct test of NFDS on pathogens evolved with ancestral microbiota, we found a significant negative linear relationship between a mutation’s change in frequency across the experiment and its frequency at the mid-point (Fig. 4C), with 58% of alleles increasing in frequency from passage 10 to 15. Pathogens evolved alone were not under NFDS (Fig. 4D). This finding suggests that when in competition with the microbiota, rare pathogen genotypes are at a selective advantage.

### Microbiota passaged in isolation facilitates pathogen virulence to a greater extent

**Figure 5:**
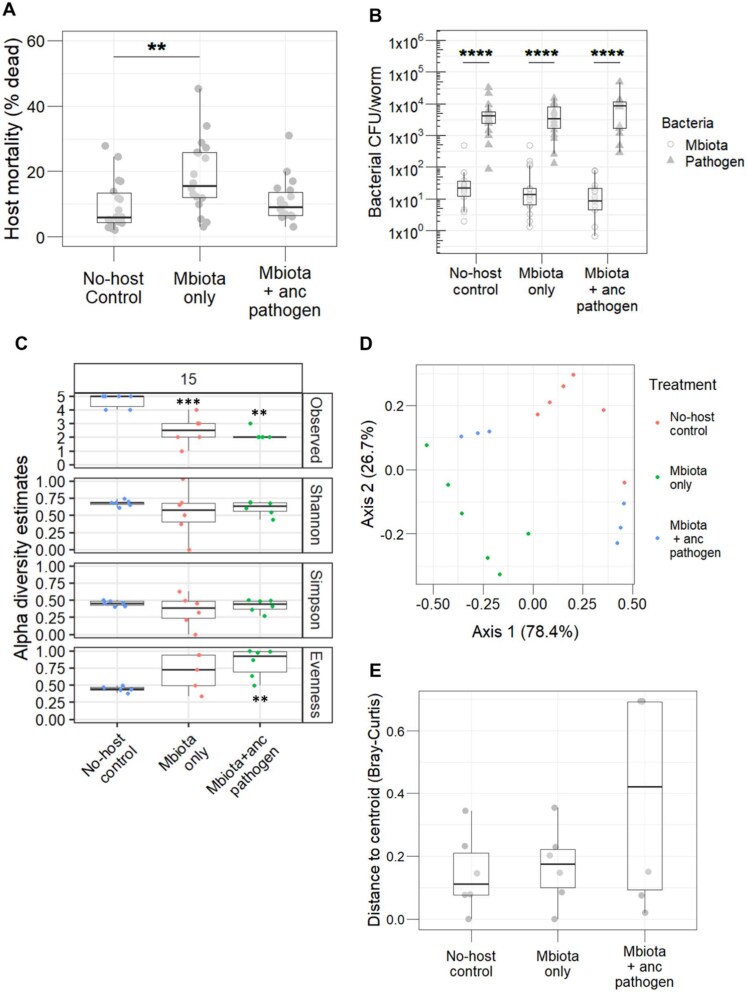
All data shown represents the 15th (final) evolved passage. (**A**) The microbiota that evolved alone facilitated the virulence of ancestral *S. aureus* to a significantly greater extent than the no-host control evolved microbiota (n = 18, Kruskal–Wallis rank sum test, df = 2, *P* = 0.01, Dunn test comparison No-host control vs Mbiota only *P* = 0.01). (**B**) Colonisation of *C. elegans* was comparable across all three evolved microbiota lineages, which all colonized the worms to a significantly lesser extent than the ancestral pathogen (n = 35-36, linear regression, *F* = 87.7, df = 5, *P* < 0.001, post-hoc comparisons using “emmeans” package: Mbiota CFU/worm vs pathogen CFU/worm *P* < 0.0001 in each evolved group). (**C**) Alpha diversity estimates of each evolved microbiota community. Asterisks indicate significant differences from the no-host control in each graph. Mbiota only has significantly reduced species richness (n = 12, linear regression, *F* = 20.6, df = 1, *P* = 0.001), as does Mbiota + anc pathogen (n = 12, linear regression, *F* = 55.4, df = 1, *P* = 0.003). Mbiota + anc pathogen also has significantly reduced evenness (n = 12, linear regression, *F* = 30.9, df = 1, *P* = 0.0002) (**D**) principal component analysis showing beta-diversity of evolved microbiota communities. Community composition differed significantly between replicates of each evolved group (PERMANOVA Bray–Curtis, *P* = 0.0001). In particular, the microbiota that evolved with ancestral pathogen is significantly more dispersed compared to the microbiota that evolved alone (PERMANOVA Bray–Curtis, *F* = 21.3, df = 1, *P* = 0.001). (**E**) Where the microbiota evolved with the ancestral pathogen, evolved communities are significantly more unstable compared to the Mbiota only group (PERMANOVA, *F* = 21.2, df = 1, *P* = 0.001).

We hypothesized that microbiota communities maintained within host populations over evolutionary time would dampen virulence of the ancestral pathogen. After passaging the microbiota through 15 host generations (Fig. 1), we found that microbiota passaged in infected hosts did not diminish virulence of the ancestral pathogen (Fig. 5A). However, microbiota passage through uninfected hosts caused significantly more host killing by ancestral *S. aureus* compared to the no-host control (Fig. 5A).

Microbiota load in the face of ancestral pathogen infection was comparable across all evolved groups (Fig. 5B – circular symbols) as was pathogen load (Fig. 5B – triangular symbols). In all cases, the ancestral pathogen was more competitive than the microbiota, colonising nematodes to a significantly greater extent than the evolved microbiota communities (Fig. 5B). RNAIII expression in the ancestral pathogen was measured in the presence of each evolved microbiota community by qRT-PCR, but no significant difference was observed (Fig. S3). Therefore, the higher level of host killing from *S. aureus* in the presence of microbiota that evolved alone was not due to higher expression of pathogen virulence factors. Microbiota passaged in uninfected hosts caused 2% average mortality, but this impact was not significantly different from mortality in the no-host control (Fig. S4). Thus, the observed increase in host mortality in the presence of *S. aureus* could not be explained by direct killing from the microbiota community maintained across generations.

### Passage in infected hosts significantly reduces microbiota community stability

**Figure 6:**
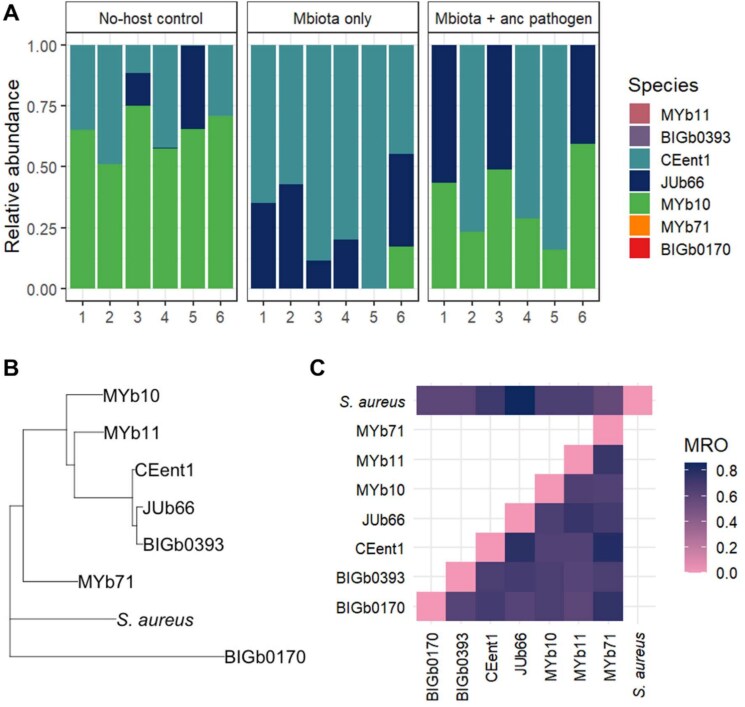
(**A**) Community composition of each replicate population within the three evolved microbiota groups at passage 15. MYb10 is significantly enriched in the microbiota that evolved in infected hosts (Wilcoxon rank sum test, *P* = 0.0002). MYb71 and BIGb0170 are enriched in the no-host control (*P* = 0.003 and 0.001, respectively). (**B**) Phylogram depicting relatedness of the seven microbiota species and *S. aureus*. (**C**) MRO between bacterial strains and pathogen pairs. JUb66 and CEent1 are phylogenetically close and have higher MRO, indicating higher competition for resources between these two species.

To examine how *S. aureus* affected the community composition of passaged microbiota populations, we conducted metagenome sequencing on microbiota communities from passage 15. We found that species richness was significantly higher in the no-host control compared to all other communities (Fig. 5C). However, species evenness in the no-host control was significantly lower (Fig. 5C). Composition of the microbiota communities differed significantly between replicates of each evolved group (Fig. 5D). MYb10 was enriched in the microbiota passaged in infected hosts, whereas MYb71 and BIGb0170 were present in the no-host control but not the other two groups. In the presence of the pathogen, the microbiota became significantly more dispersed compared to all other microbiota communities (Fig. 5E). Specifically, pathogen infection drove the microbiota to form two distinct profiles, dominated either by MYb10 and JUb66, or MYb10 and CEent1 (Figs. 5D & 6A).

Pathogen infection altered competitive dynamics within the microbiota. Having been passaged in hosts infected by the ancestral pathogen, strains CEent1 and JUb66 were never found to co-exist at passage 15. In contrast, MYb10 was always maintained in these replicates (Fig. 6A). The opposite dynamic was seen in replicates of the microbiota evolved in uninfected hosts (Fig. 6A), whereby CEent1 and JUb66 regularly co-existed, but MYb10 was mostly absent from the community. We found that CEent1 and JUb66 were phylogenetically closely related (Fig. 6B) and had a high degree of MRO (Fig. 6C). A high degree of resource overlap, as found between CEent1 and JUb66, indicates strong competitive interactions for nutritional resources. Manipulation of the in vivo nutritional environment is needed to further characterize these interactions.

### Predicted cross-feeding interactions between host microbiota and *S. aureus*

We investigated cross-feeding interactions between microbiota components to identify which resources were the source of competition. BIGb0170 and BIGb0393, both as metabolic donors to *S. aureus* of Fe^2+^ and O_2_, have high SMETANA scores of 1 (a SMETANA score of 1 indicates absolute certainty on the cross-feeding interaction). CEent1 can serve as a donor of copper and Fe^2+^ to *S. aureus* (SMETANA score = 1). MYb10 and MYb71 can donate phosphate and copper to *S. aureus*, respectively (SMETANA scores = 1). Conversely, *S. aureus* can donate O_2_ to MYb10 and MYb71 (SMETANA scores = 1), as well as donating phosphate to MYb71 (SMETANA score = 1) and L-Cysteine to MYb10 (SMETANA score = 0.9). Within the microbiota, cross-feeding interactions were much weaker, shown by smaller SMETANA scores for microbe pairs (see supplementary file 1). In particular, we found that JUb66 and CEent1 were exclusively donors of metabolites to other microbiota members, which might exacerbate their competition for nutrients in *S. aureus*–infected hosts.

### Short-term pathogen evolution

We tested whether transplanting a host-adapted microbiota community could alter the trajectory of within-host pathogen evolution over a short timescale. We introduced microbiota evolved in uninfected hosts (from passage 10) into the group in which pathogens were evolving alone. Both microbiota and pathogen in this newly created group, likewise consisting of six replicates, evolved for the remaining five passages alongside the original evolving groups (Fig. S5). Comparing the virulence of *S. aureus* at passage 15 between this group and the original pathogen that evolved alone, we did not find any significant differences in host mortality from infection (Fig. S6). Introduction of microbiota adapted to uninfected hosts therefore did not significantly alter the trajectory of *S. aureus* virulence on this time scale.

## Discussion

Most microbes face constant warfare with others [55, 56], which may be heightened during infection by a pathogen. Competition between pathogens [57], or within microbial symbiont communities [55], can be a major selective force shaping virulence. Here, upon initial invasion of nematode hosts, *S. aureus* displaced resident microbiota (in terms of number and diversity) worsening disease outcome, comparable to results observed with other bacterial pathogens [58]. This finding contrasted with *in vitro* outcomes, whereby the presence of the microbiota significantly reduced pathogen accumulation (Fig. 2D). Gene expression patterns of *S. aureus* have previously been observed to differ considerably between *in vitro* and *in vivo* conditions [59, 60] and various factors could be responsible for the difference observed in our model. The *in vivo* environment of the worm gut is likely resource-limited (with nutrient availability being highly dependent on diet [61, 62]), and spatial structures in the gut may also affect bacterial colonisation [63, 64]. In the context of the nematode gut, *S. aureus* is likely better able to adhere to host epithelial cells and acquire nutrients from the host (as observed in^59^), outcompeting host microbiota. Further research from this system also illustrates that microbiota-colonized hosts have increased expression of immunity genes and variable expression of stress response genes during infection, which may also contribute to worsening disease outcomes [65]. In *in vitro* conditions, the nutrient-rich, unstructured environment may favour microbiota members, potentially facilitating the production of antimicrobial peptides or resource competition detrimental to pathogen growth.

We found the evolutionary and ecological paths of pathogens and host microbiota, respectively, varied depending upon the competitive opportunities within nematode hosts. In our experiment, competition with host microbiota allowed for the maintenance of pathogen virulence over evolutionary time at levels seen in no-host controls (Fig. 3). The consistent dominance and high virulence of *S. aureus* in microbiota-colonized hosts could be likened to “super-infections” whereby a virulent pathogen can take over a host already infected by a less virulent one. Superinfections can maintain highly virulent pathogen strains across evolutionary time if virulence is equated with within-host competitive advantage [15]. The high pathogen load and high level of host harm caused by infection in nematodes with natural microbiota may thus limit any movement in optimal virulence. However, we found competition with host microbiota caused a burst of pathogen molecular evolution and diversification early on (Fig. 4). Superinfection can lead to complex evolutionary dynamics involving heteroclinic (oscillating) cycles of genetic mutations within a population [15], which can be driven by NFDS generated from species interactions [66, 67]. We found that pathogens evolving amidst nematode microbiota were more likely to experience NFDS at the genomic level. *S. aureus* is a relatively clonal organism, so point mutations are the more common source of new alleles in this species [68]. Previous work from our lab shows that few large-effect mutations can often be responsible for changes in virulence across a diversity of microbial pathogens in worms [69–71]. Work in in vivo microbial experimental evolution has also shown that virulence is often a polygenic trait [69–71], thus it is unlikely a single allele would be responsible for virulence changes in this experiment. Greater genetic divergence was observed among replicate evolved populations in the pathogen that evolved alongside microbiota, whereas pathogen populations evolving in isolation grouped together genotypically. NFDS has been found to similarly cause population diversification in other microbial species interactions [72, 73].

Pathogen evolution in isolation favoured a two-fold increase in host mortality from infection compared to the no-host controls (Fig. 3A). This outcome is common in serial passage experiments, in which transmission is guaranteed by experimental design, therefore reducing the cost of high virulence [74]. Passage of pathogens in genetically homogeneous host populations (as in the present study) is also hypothesized to select for specialisation on exploiting those hosts [75, 76]. In our experiment, passage in homogeneous host populations (without microbiomes) favoured selection on traits that enabled better colonisation of the host environment, many of which (e.g. adhesion, immune evasion, nutrient acquisition) are also key aspects of *S. aureus* virulence [30, 77]. Hosts with microbiomes may alternatively limit that specialisation, as shown in our study. Whether this evolutionary maintenance of virulence is consistent in other animal-pathogen systems where pathogens similarly suppress microbiota or exploit dysbiotic communities (e.g. *Clostridioides difficile*, *Salmonella Typhimurium*) [78, 79] remains to be explored.

The increase in virulence in the pathogen that evolved alone could be due to selection on a combination of virulence factors. Namely, we found that biofilm production and the expression of *agr,* a global virulence regulator, were higher in the pathogen that evolved alone compared to the no-host control. The ability to form a biofilm can improve a pathogen’s ability to colonize host tissues and resist immune clearance [52, 80] This trait has been a target of selection during evolution experiments and is an important aspect of pathogen virulence in *C. elegans* [81]*. agr* has been shown to contribute to staphylococcal infection in a range of host models [34, 82, 83], with *agr*-deficient mutants exhibiting reduced virulence [82, 84]. Selection on *agr* has been revealed to impact virulence evolution in *S. aureus* [85]*.* It controls expression of a range of secreted toxins, including the alpha, beta, and delta haemolysins, toxic shock syndrome toxin, and staphylokinase [46]. High *agr* activity is often associated with reduced biofilm formation – or dispersion and detachment of staphylococcal biofilms [80, 86]. The fact that quorum-sensing activates *agr* at high cell densities suggests its activity may vary in our system as infection progresses [87]. Perhaps a biofilm forms in the host gut initially, with *agr* expression induced as pathogen densities increase, allowing cells to detach from biofilms and disperse within the site of infection [87].

Pathogen presence during in vivo microbiota passages strongly shifted community dynamics (Fig. 6). Infection is often indirectly associated with dysbiosis of the microbiota [88, 89]. Here, using metabolic modelling analysis, we found a direct link between *S. aureus* infection and increased potential nutrient competition between resident microbes, with destabilising effects on community composition over evolutionary time. Whilst *Acinetobacter gouilliae* (MYb10) can be a recipient of nutrients during cross-feeding with *S. aureus,* and thus maintained in every replicate, pathogen infection prevented the coexistence of two closely related microbiome species. We observed the stochastic competitive exclusion of either *Enterobacter hormaechei* (CEent1) or *Lelliottia amnigena* (JUb66) within all replicates of microbiota communities passaged with the pathogen. Similar dynamics have been observed with *Clostridioides difficile*, whereby microbiota community structure is altered during infection due to changes induced in the nutritional landscape [90]. Our findings fit with the Anna Karenina Principle, whereby dysbiotic microbiota communities differ more in community composition compared to healthy (eubiotic) communities [91]. We find that destabilization arises in infected hosts across both ecological and evolutionary time. This pattern suggests that infected hosts are less capable of regulating their microbiota community [91, 92], with microbiota composition lacking a steady state (i.e. being more dispersed) in the face of pathogen invasion [93, 94] (Fig. 5E).

We found that higher disease severity was caused when worms were colonized by microbiota passaged in isolation and infected by ancestral pathogens, compared to when worms were colonized by control microbiota (Fig. 5). A parallel can be drawn between our findings and the high levels of virulence often observed when a pathogen jumps into a new host. *S. aureus* in particular has a wide host range [27, 28] and is known to jump between animal species [95–97]. Upon invading a new host, *S. aureus* will also encounter a new microbiota with which it does not share an evolutionary history. Competition with the resident microbiota community might therefore contribute to more severe disease outcomes from novel *S. aureus* infection. Having previously travelled on separate evolutionary paths, we found invading (ancestral) *S. aureus* and host-adapted resident microbiota exhibit greater antagonism upon first meeting. Considerable research has been conducted to characterize interactions between *S. aureus* and host microbiota communities [98–100]. Future investigations seeking to understand disease severity from *S. aureus* infections, and other generalist pathogens with spillover potential [101, 102], should consider the role of competition with host microbiota during these novel interactions.

Patterns of infectious disease distribution and transmission are predicted to undergo considerable changes in coming years [103]. Climate change, biodiversity loss, and associated human activities are providing more opportunities for pathogens to jump between animal species and humans [104]. Our study reveals that when pathogens infect new hosts, competitive dominance over microbiota sparks pathogen molecular diversification, whereas virulence evolution is limited. Similar pathogen genome dynamics have been uncovered during coevolution whereby changes in host genetic background, but not microbiome communities, generates fluctuating selection [105]. Moreover, the ecological factors imposing selection on microbiomes are increasingly being uncovered [106, 107]. For host microbiomes herein, infection by a novel pathogen was a perpetually destabilising force. Such variability and stochasticity in microbiome composition highlights the difficulty of predicting infection outcome during epidemics of new pathogens. Ultimately, understanding the mechanisms of host microbiota-pathogen interactions may help explain infection outcomes now and across evolutionary time both in wildlife and humans.

## Supplementary Material

25-4-8_Supplementary_material_and_figures_wraf071

Phenotypic_data_for_evo_exp_paper_wraf071

Supplementary_file_1-smetana_scores_wraf071

Supplementary_phenotypic_data_wraf071

## Data Availability

Phenotypic data are provided as an Excel file alongside the submitted manuscript. Genomic data (Accession number PRJNA1055888) can be viewed at this link https://www.ncbi.nlm.nih.gov/bioproject/PRJNA1055888.
