# Supplementary material for: Within-host competition causes pathogen molecular evolution and perpetual microbiota dysbiosis
Source: ISME J. 2025 Apr 17;19(1):wraf071. doi: 10.1093/ismejo/wraf071 (PMC12066030; doi:10.1093/ismejo/wraf071)

**Supplementary material**

1. The effect of evolved pathogens on microbiota community assembly is host-mediated

We investigated how the evolved pathogens’ competitive colonisation advantage affected microbiota community structure. We conducted 16S rRNA gene sequencing on the ancestral microbial communities in nematodes infected for 24 hours with each evolved pathogen population (passage 15). We found that alpha diversity and composition of the ancestral microbiota community did not differ across each of the evolved pathogens, within in vitro or in vivo samples (figs S7-8). Composition and alpha diversity differed significantly between paired in vitro and in vivo samples however (fig S7), indicating a strong host-mediated effect of infection on microbiota community assembly, as observed in the ancestral state. In vivo, MYb10 dominated across all groups, followed by MYb71 and MYb11. BIGb393 and BIGb0170 were mostly excluded from the community, while CEent1 and JUb66 were consistently present at low abundances (fig S8a). In vitro, CEent1 dominated, making up 75% or more of the community in each replicate. BIGb393 and MYb71 were mostly excluded, while MYb11 was present at low abundance in around two thirds of replicates. MYb10 was present in most replicates at low abundance. JUb66 and BIGb0170 were consistently present across replicates but also at low abundances (fig S8b).

2. Analysis of genes under selection

We used the genome comparison tool ACT^1^, BLAST searches for homologous proteins and literature searches to ascertain the function of each gene under selection. Genes were categorised by broad functional category (metabolism, virulence or adherence) based on their primary function. Genes under selection in the pathogen that evolved alone with possible links to staphylococcal virulence include *purR* (*pur* operon repressor), *icaC* (polysaccharide intercellular adhesin biosynthesis/export protein IcaC), *gltD* (glutamate synthase, SAS_RS02230), *clfA* (clumping factor A), and *dltB* (PG:teichoic acid D‑alanyltransferase), along with an intergenic region close to the *mnh* operon (intergenic between SAS_RS03200 and SAS_RS03205). However, no clear pattern emerged to indicate a genomic basis for the change in virulence of pathogen populations in this group, with different genes under selection in each replicate. These results suggest that virulence under selection in our experiment is based on multiple genes, as found in other pathogens with broad host ranges^2–4^. A number of SNPs were located in genes with links to biofilm formation. Where the pathogen evolved with ancestral microbiota, 4 SNPs across 3 replicates had links to biofilm formation, along with 4 SNPs across 3 replicates in the pathogen that evolved alone (see table S1). In the latter, these genes were *icaC*, *gltD*, *clfA* and an intergenic mutation in the ribosome binding site upstream of the gene SAS_RS08335, encoding *apt*.

3. Analysis of nucleotide diversity across experimental time points

We calculated the genetic distance of replicates in each group from the ancestor. No differences were observed in distance from the ancestor, indicating no difference in the overall rate of evolution between the two groups (fig S9a). We also analysed nucleotide diversity across passages 10 and 15 between the two groups and found no significant difference (fig S9b). The frequency of mutations ranged from 5-100%, of which there were generally more nonsynonymous than synonymous SNPs across treatments at each time point (figs S9c-e). All mutations are included in figure S9d, not just those that are targets of selection. Across populations, there was variation in terms of the frequency of nonsynonymous vs. synonymous SNPs. For example, most mutations in pathogen only P15 population six were at <25% frequency, whereas populations one and three of the same treatment and time point had many mutations at >50% frequency (figs S9f-g). Across the genome, there were more nonsynonymous than synonymous SNPs (fig S9h). One region in the genome exhibited a high number of mutations at moderate frequencies, but because this occurred across all treatments at both time points, these mutations likely arose from general passaging and not any particular treatment. Table S2 breaks down the number of SNPs in genes and intergenic regions for each population within each treatment across the two time points. On average, each treatment had similar densities of genic mutations by passage 15.

**Methods**

**16S rRNA gene sequencing of microbiota**

For 16S rRNA gene sequencing, bacterial DNA was extracted from crushed worms using a ZymoBIOMICS DNA Miniprep kit (Zymo) according to the manufacturer’s protocol. The V3-V4 regions of bacterial 16S rRNA genes were amplified using the universal primer pair 341F (5’-CCTACGGGNGGCWGCAG-3’) and 805R primer (5’-GACTACHVGGGTATCTAATCC-3’). PCR amplicons were sequenced on the Miseq (Illumina) platform using 2 x 300bp v3 chemistry by the Integrated Microbiome Resource at Dalhousie University, Canada. FastQC^5^ and MultiQC^6^ were used for initial visualization of read quality, primers were removed using Cutadapt^7^. Paired-end reads were joined using vsearch^8^. All low-quality reads were then filtered using default quality thresholds before starting the Deblur^9^ workflow to denoise and classify sequences into amplicon sequence variants (ASVs). Trimming length was determined as 400 bp after manually viewing the quality plot. As full-length 16S rRNA gene sequences for the seven microbiota species were well- characterized^10^, the obtained sequencing reads were processed through the closed-reference OTU picking pipeline in QIIME2^11^. To build the reference, full-length 16S rRNA gene sequences were downloaded for the seven microbiota species and converted to a qza-formatted reference file for processing by QIIME2. Taxonomy of the resolved ASVs was assigned by clustering ASVs to the customized reference with 99% similarity thresholds.

The relative abundance table was rarefied to the minimum sample size and alpha-diversity indices – Richness, Shannon index, Simpson index and Evenness were computed using the R package phyloseq. For beta diversity, Bray-Curtis dissimilarity was calculated based on rarefied relative abundance, using the R package phyloseq. Permutational analysis of variance (PERMANOVA) was conducted with 9999 replications on each distance metric to evaluate differences in the microbiome structure and composition between treatments using the R package vegan^12^. Microbiome dispersion was calculated using the betadisper function in R. Differences in microbiome dispersion between different treatments were tested using a permutation test.

16S rRNA gene sequences were downloaded for each of the seven microbiota species and the pathogen. Multiple sequence alignment (MSA) was performed using MUSCLE^13^ and the MSA was used to reconstruct a maximum-likelihood tree in IQTREE (v1.6.11)^14^. Tree reconstruction was performed with 1000 ultrafast bootstrap replicates and the SH-like approximate likelihood ratio test ("-bb 1000 -alrt 1000”). The best-fit model “TN+F+G4” was selected by ModelFinder^15^ based on the Bayesian information criterion (BIC). The phylogenetic distance matrix was generated from the consensus tree using the ‘cophenetic’ function in R.

**Calculation of nucleotide diversity**

We calculated the nucleotide diversity across the entire pathogen genome for each replicate population to quantify the genetic diversity that arose from the ancestral clone. Nucleotide diversity, or pi, is the average number of nucleotide differences between all possible pairs of individuals in the population. We used the software PoPoolation^16^ to calculate nucleotide diversity (Tajima’s pi) using a sliding window analysis across the entire genome of evolved S. aureus. Each window size was 500bp with a step-size of 250bp. We calculated the mean of all windows with a positive value within each population, then the mean of all populations within each treatment across time.

**References**

1. Carver, T. J. *et al.* ACT: the Artemis comparison tool. *Bioinformatics* **21**, 3422–3423 (2005).

2. Chen, H. *et al.* Polygenic virulence factors involved in pathogenesis of 1997 Hong Kong H5N1 influenza viruses in mice. *Virus Res* **128**, 159–163 (2007).

3. Caseys, C. *et al.* Quantitative interactions: the disease outcome of Botrytis cinerea across the plant kingdom. *G3 Genes|Genomes|Genetics* **11**, jkab175 (2021).

4. Le Clec’h, W. *et al.* Genetic architecture of transmission stage production and virulence in schistosome parasites. *Virulence* **12**, 1508–1526 (2021).

5. Wingett, S. W. & Andrews, S. FastQ Screen: A tool for multi-genome mapping and quality control. *F1000Res* **7**, 1338 (2018).

6. Ewels, P., Magnusson, M., Lundin, S. & Käller, M. MultiQC: summarize analysis results for multiple tools and samples in a single report. *Bioinformatics* **32**, 3047–3048 (2016).

7. Martin, M. Cutadapt removes adapter sequences from high-throughput sequencing reads. *EMBnet.journal* **17**, 10–12 (2011).

8. Rognes, T., Flouri, T., Nichols, B., Quince, C. & Mahé, F. VSEARCH: a versatile open source tool for metagenomics. *PeerJ* **4**, e2584 (2016).

9. Amir, A. *et al.* Deblur Rapidly Resolves Single-Nucleotide Community Sequence Patterns. *mSystems* **2**, e00191-16 (2017).

10. Dirksen, P. *et al.* CeMbio - The Caenorhabditis elegans Microbiome Resource. *G3 Genes|Genomes|Genetics* **10**, 3025–3039 (2020).

11. Bolyen, E. *et al.* Reproducible, interactive, scalable and extensible microbiome data science using QIIME 2. *Nat Biotechnol* **37**, 852–857 (2019).

12. Oksanen et al. _vegan: Community Ecology Package_. R package version 2.6-4. (2022).

13. Madeira, F. *et al.* Search and sequence analysis tools services from EMBL-EBI in 2022. *Nucleic Acids Res* **50**, W276–W279 (2022).

14. Chernomor, O., von Haeseler, A. & Minh, B. Q. Terrace Aware Data Structure for Phylogenomic Inference from Supermatrices. *Systematic Biology* **65**, 997–1008 (2016).

15. Kalyaanamoorthy, S., Minh, B. Q., Wong, T. K. F., von Haeseler, A. & Jermiin, L. S. ModelFinder: fast model selection for accurate phylogenetic estimates. *Nat Methods* **14**, 587–589 (2017).

16. Kofler, R. *et al.* PoPoolation: A Toolbox for Population Genetic Analysis of Next Generation Sequencing Data from Pooled Individuals. *PLOS ONE* **6**, e15925 (2011).

**Supplementary figures**


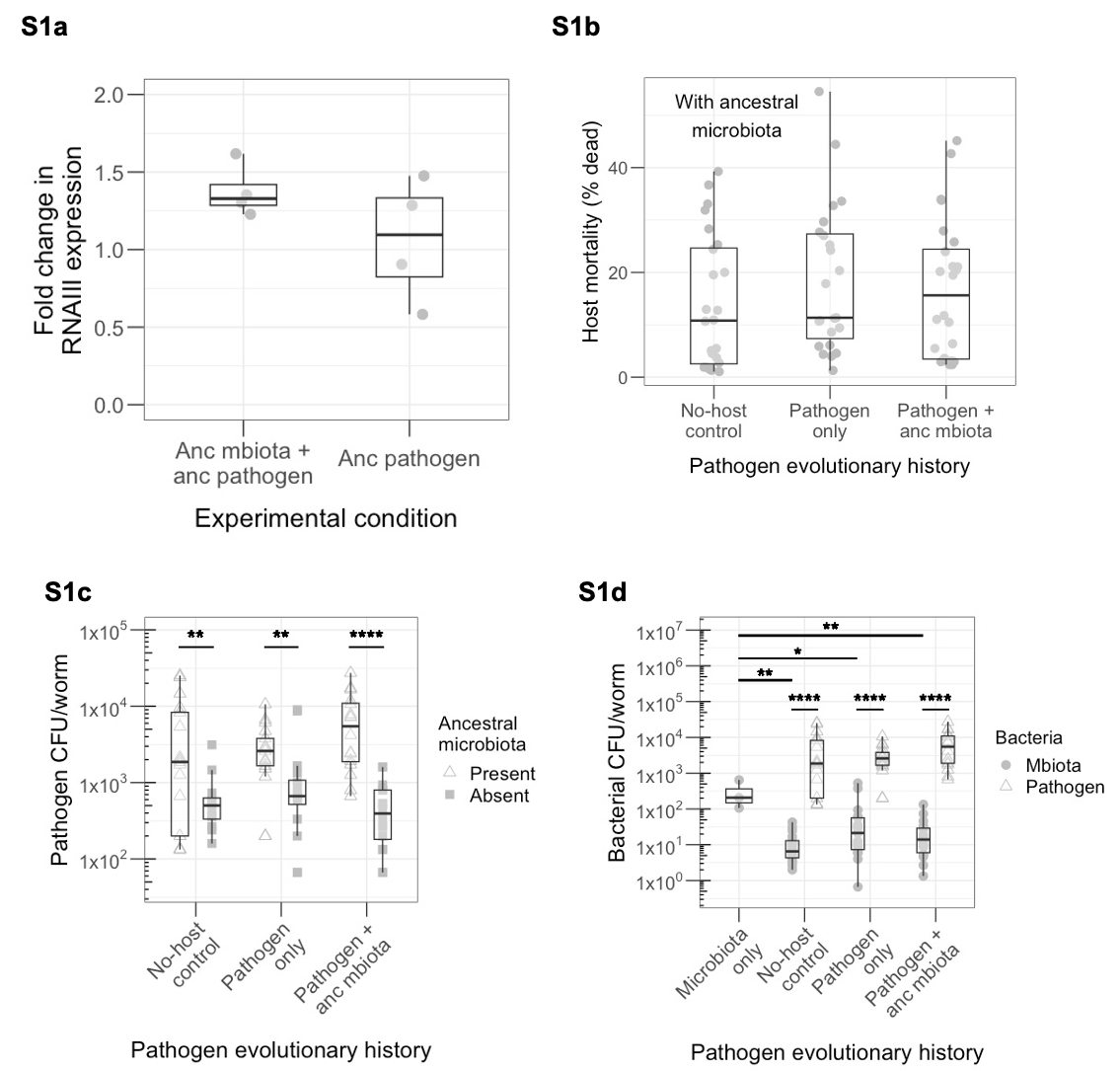


**Supplementary figure 1. S1a)** No difference in in vivo RNAIII expression of the ancestral pathogen in the presence and absence of the ancestral microbiota (n=4, Welch Two-sample T test, *P*=0.22). **S1b)** In the presence of ancestral microbiota, there is no difference in host mortality from each evolved pathogen (n=23-24, Kruskal Wallis rank sum test, df=2, *P*=0.373). **S1c)** All evolved pathogens colonise *C. elegans* significantly better in the presence, compared to the absence, of ancestral microbiota (n=12-17, linear regression using log10 of raw data, *F*=8.6, df=5, *P*<0.0001, pairwise comparisons of each group in the presence vs absence of ancestral microbiota, using ‘emmeans’ package: No-host control *P*=0.023, Pathogen only *P*<0.0001, Pathogen + anc mbiota *P*=0.014) **S1d)** Colonisation of *C. elegans* by the ancestral microbiota is significantly reduced by the presence of all evolved pathogens compared to the microbiota only (n=3 for microbiota only control, n=16-18 for all other groups, linear regression using log10 of raw data, *F*=64.5, df=6, *P*<0.0001, pairwise comparisons between mbiota CFU/worm using ‘emmeans’ package: Microbiota only vs No-host control *P*=0.0006, Microbiota only vs Pathogen only *P*=0.025, Microbiota only vs Pathogen + anc mbiota *P*=0.005), and all evolved pathogens colonise *C. elegans* to a significantly greater extent than the microbiota (n=16-18, linear regression using log10 of raw data, *F*=64.5, df=6, *P*<0.0001, pairwise comparisons between pathogen CFU/worm and mbiota CFU/worm in each evolved group: all *P*<0.0001).


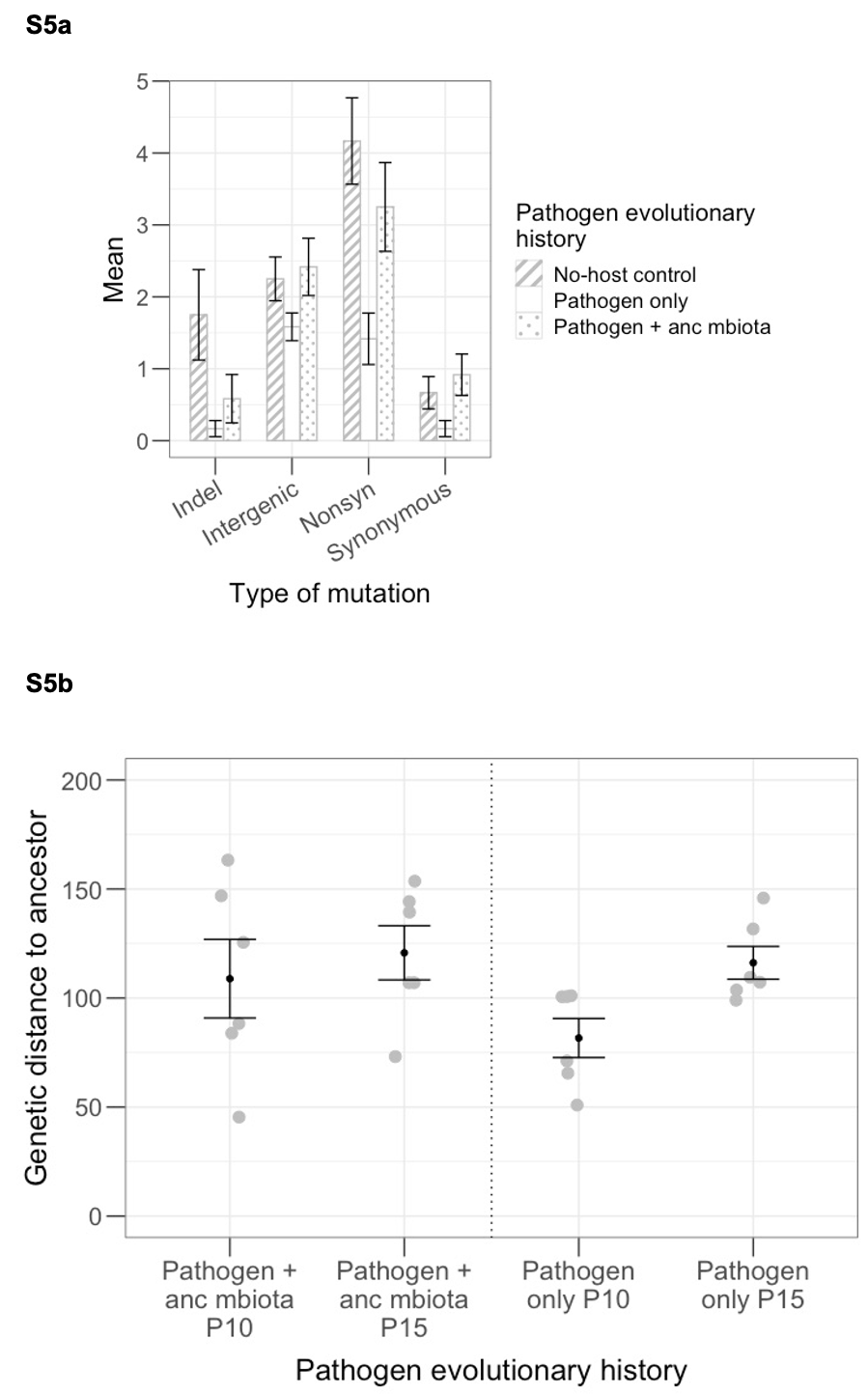


**S2a**

**S2b**


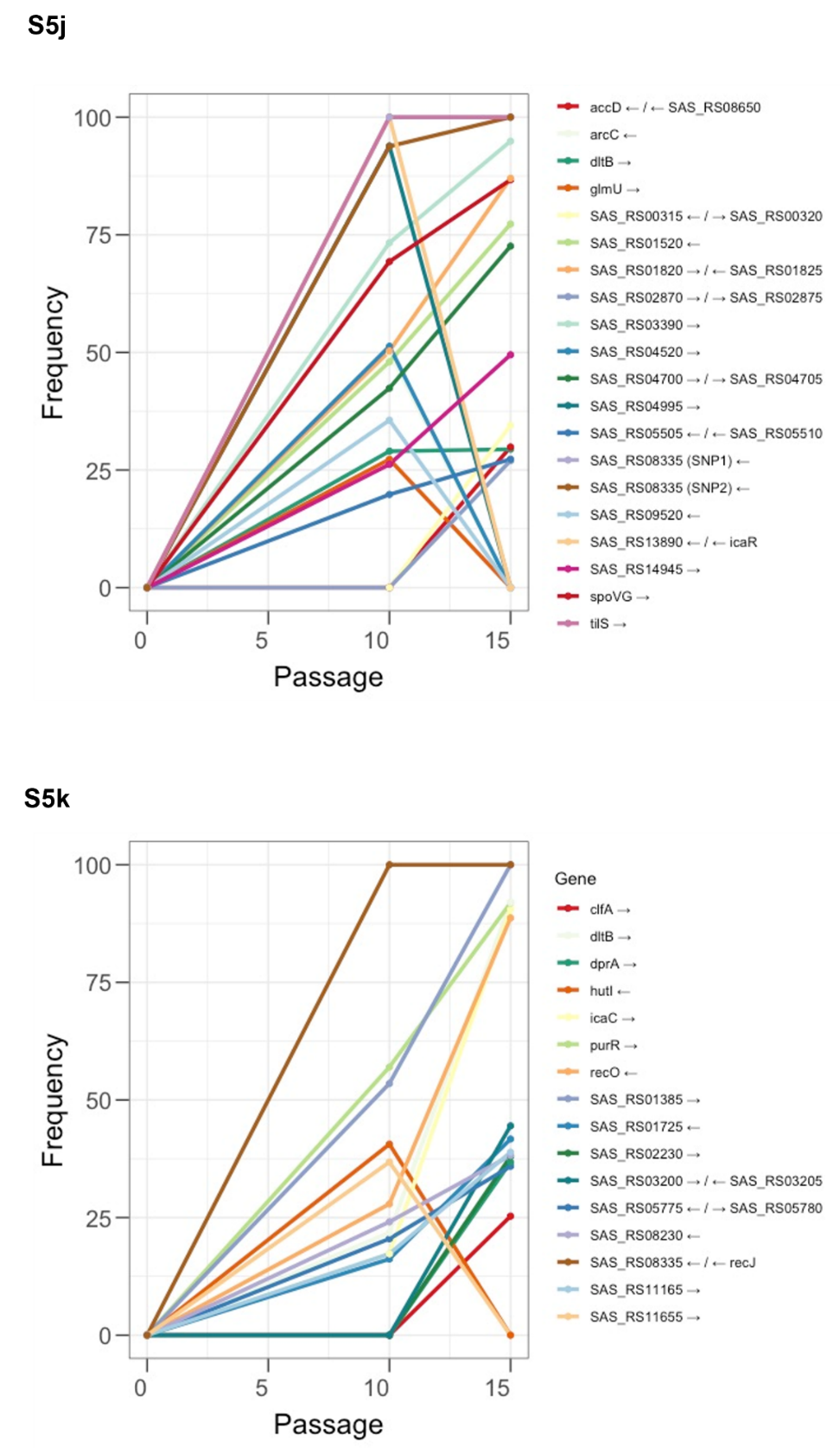


**S2c**

**S2d**

**Supplementary figure 2. S2a)** Significantly fewer non-synonymous mutations (n=12, ANOVA *F*=6.8, df=2, *P*=0.004, TukeyHSD comparison between No-host control and Pathogen only *P*=0.003) and indels (n=12, Kruskal Wallis rank sum test df=2, *P*=0.02, Dunn test comparison between No-host control and Pathogen only *P*=0.02) account for the difference in total number of mutations in clones of the ‘Pathogen only’ group. **S2b)** Genetic distance from the ancestor of each replicate population within each evolutionary group at generations 10 and 15. No significant differences between groups (n=6, ANOVA *F*=2.0, df=3, *P*=0.14). **S2c)** Mutation frequency at passages 10 and 15 for replicates of the pathogen that evolved with ancestral microbiota. **S2d)** Mutation frequency at passages 10 and 15 for replicates of the pathogen that evolved in isolation.


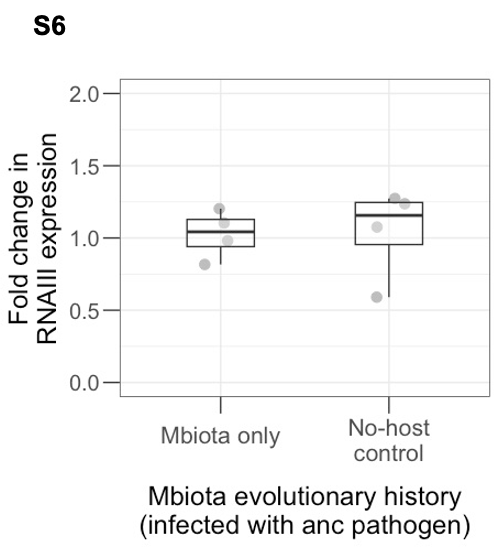


**S3**

**Supplementary figure 3.** No difference in *in vivo* RNAIII expression of the ancestral pathogen in the presence of the microbiota that evolved alone and the no-host control evolved community (n=4, Welch Two-sample T test *P*=0.924).


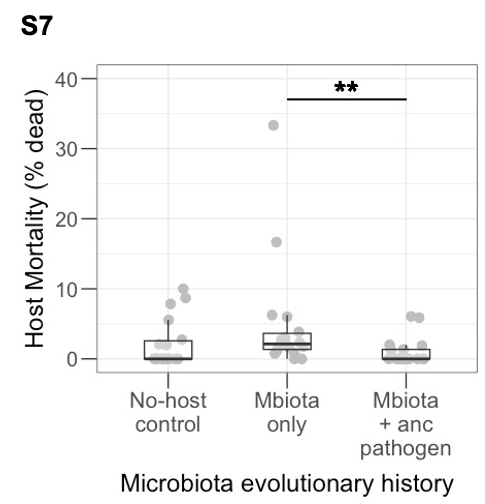


**S4**

**Supplementary figure 4.** When colonised with only the evolved microbiota lineages and no pathogen, the microbiota that evolved alone caused slightly higher mortality in *C. elegans*, which was significantly different from the Mbiota + anc pathogen, but not significantly different to the no-host control (n=17-18, Kruskal Wallis rank sum test, df=2, *P*=0.01, Dunn test comparisons: No-host control vs Mbiota only *P*=0.099, Mbiota only vs Mbiota + anc pathogen *P*=0.012, No-host control vs Mbiota + anc pathogen *P*=0.29).


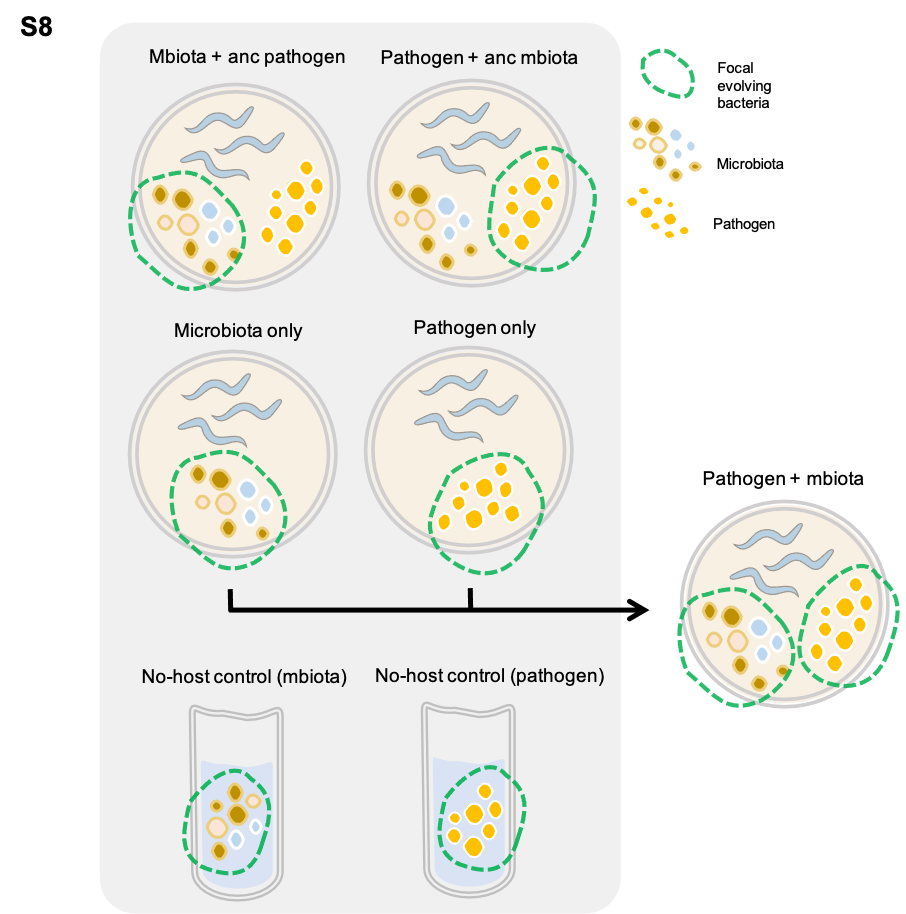


**S5**

**Supplementary figure 5.** Overview of newly created evolving group at passage 10, in which the evolved ‘Microbiota only’ community was introduced to duplicates of the ‘Pathogen only’ group. This new group evolved in parallel to the original 6 groups for the remaining 5 passages of the evolution experiment.


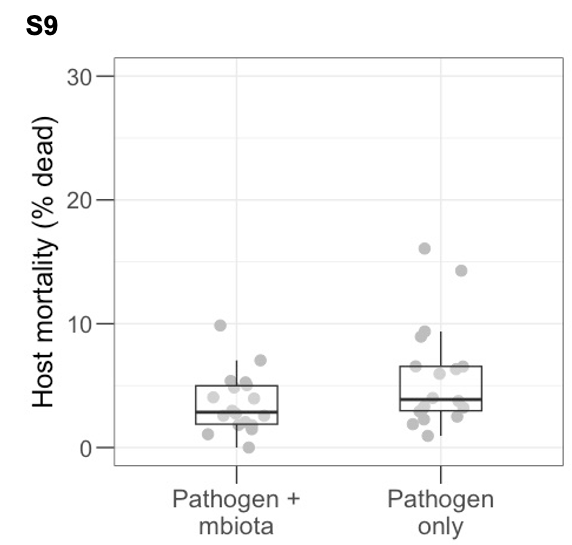


**S6**

**Supplementary figure 6.** When the evolved ‘Microbiota only’ community was introduced to duplicates of the ‘Pathogen only’ group at passage 10, no significant difference in virulence was observed at passage 15 between the respective pathogens from these two groups (n=18, Kruskal Wallis rank sum test, df=1, *P*=0.11).

**S7**


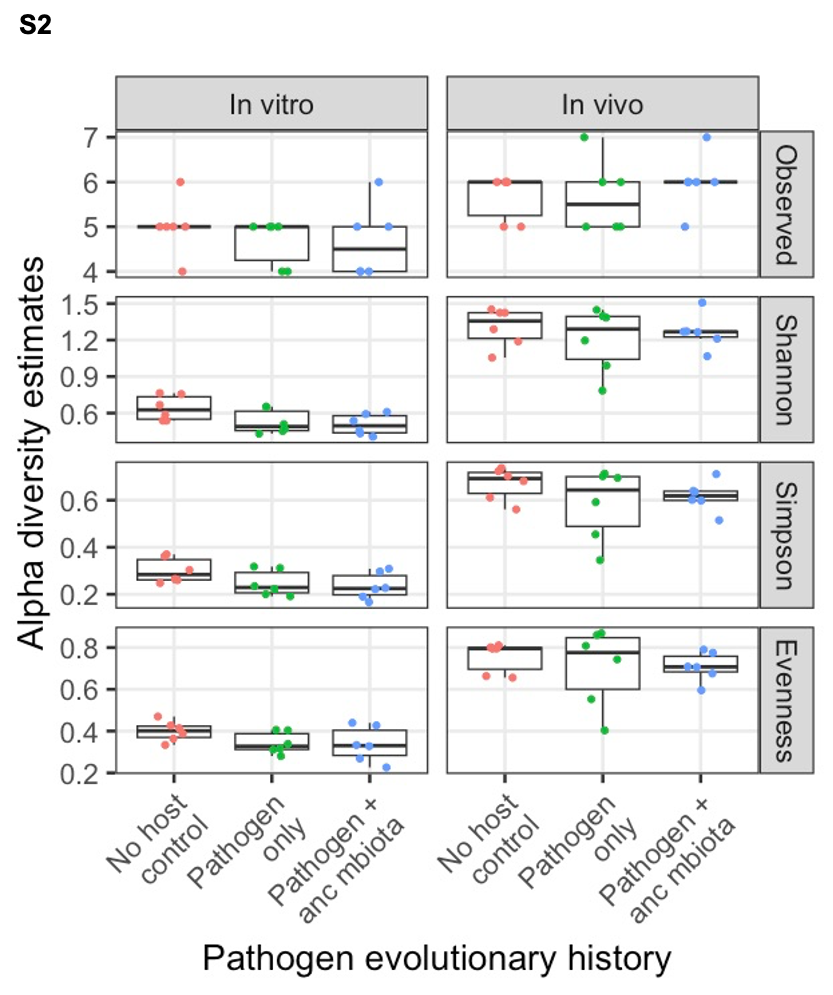


**Supplementary figure 7.** No significant differences in alpha diversity of the ancestral microbiota community in the presence of each evolved pathogen in vitro, based on 16S rRNA gene sequencing (n=6, Kruskal Wallis rank sum test for Observed diversity *P*=0.57, ANOVA for Shannon index *P*=0.06, for Simpson *P*=0.12 and for Evenness *P*=0.21) or in vivo (n=6, Kruskal Wallis rank sum test for Observed diversity *P*=0.57, for Simpson *P*=0.37 and for Evenness *P*=0.53, ANOVA for Shannon index *P*=0.65).


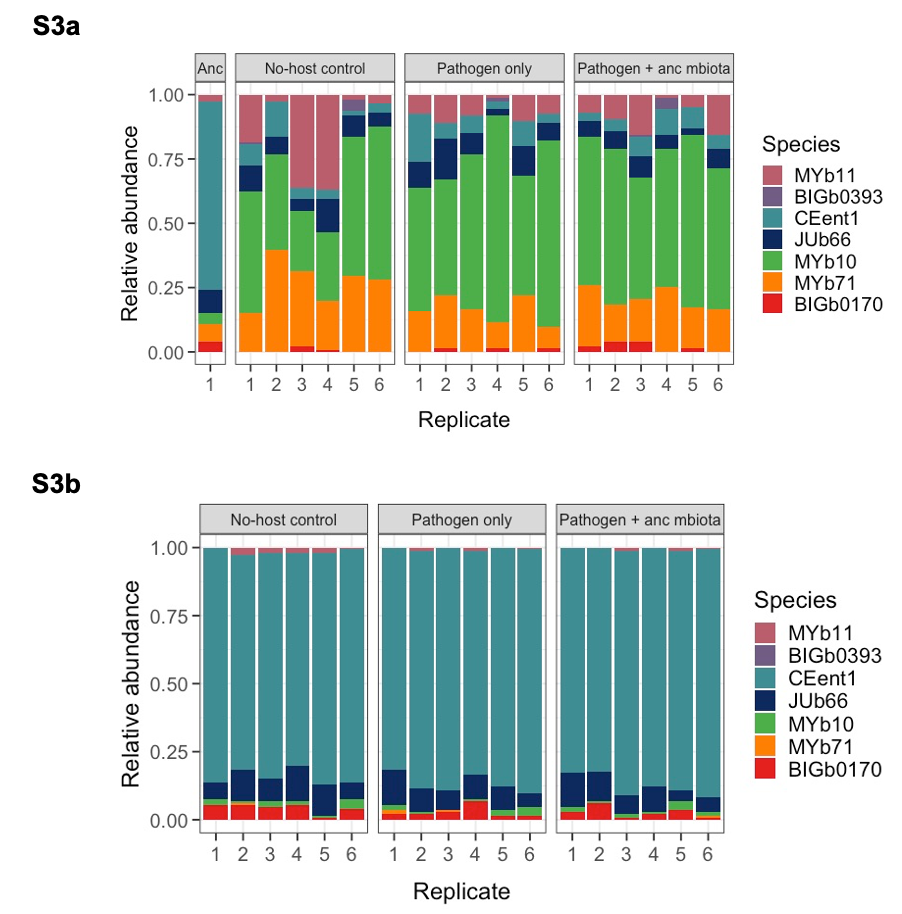


**S8a**

**S8b**

**Supplementary figure 8. S8a)** Composition data from 16S rRNA gene sequencing of in vivo samples. (PERMANOVA Bray Curtis pseudo-*F*=2.5, *P*=0.047, pairwise comparison yields significant difference between ‘Pathogen only’ and ‘No-host control’, pseudo-*F*=3.2, *P*=0.051). **S8b)** Composition data from 16S rRNA gene sequencing of in vitro samples. No significant differences in community composition across evolved groups (PERMANOVA Bray-Curtis pseudo-*F*=1.5, *P*=0.2176).


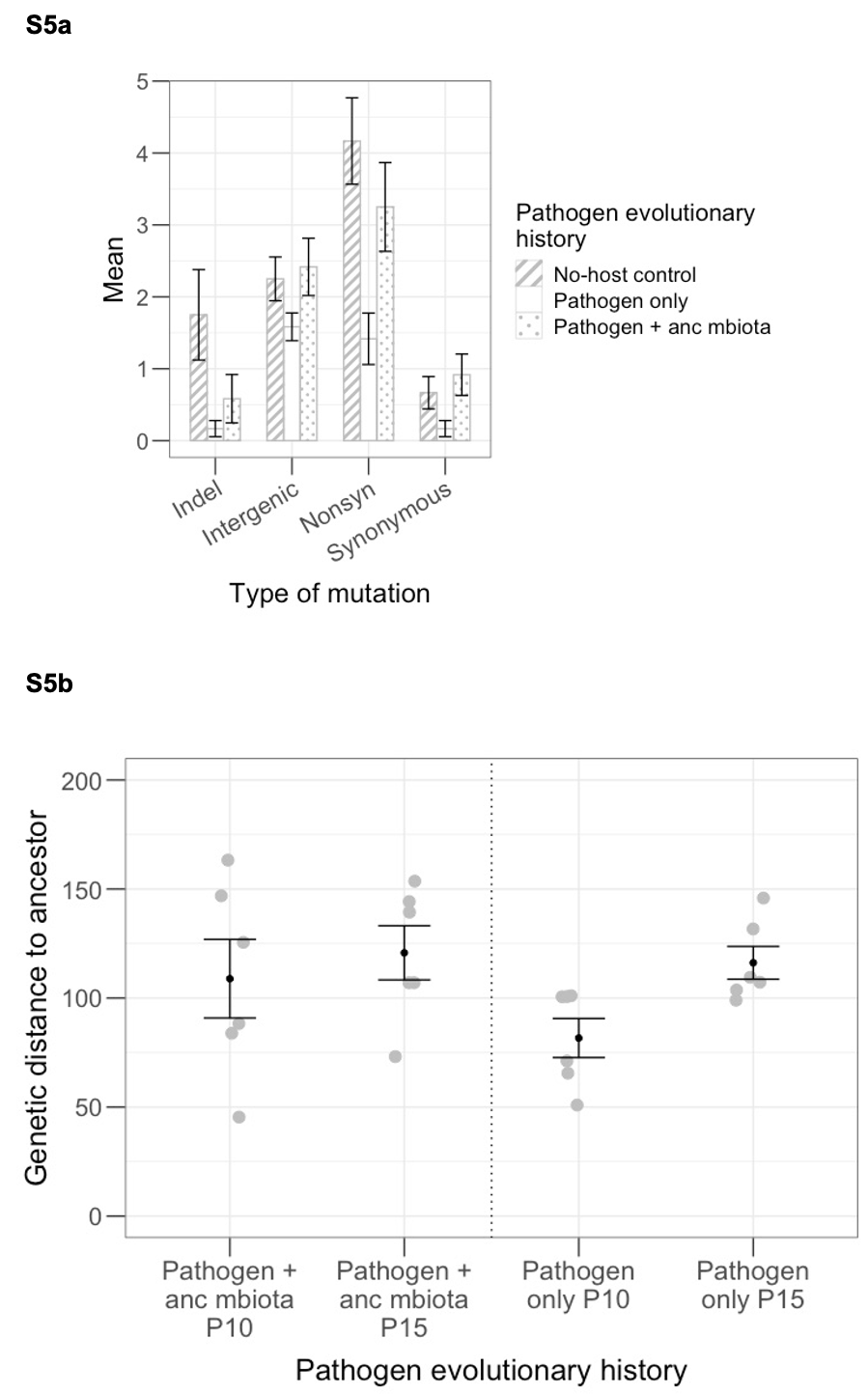


**S9a**

**Supplementary figure 9. S9a)** Genetic distance from the ancestor of each replicate population within each evolutionary group at generations 10 and 15. No significant differences between groups (n=6, ANOVA *F*=2.0, df=3, *P*=0.14).

**S9b**

**S9b)** Nucleotide diversity averaged across genome for each treatment group. No significant differences in nucleotide diversity were found between treatments or within treatments over time (comparison of all four groups - P10 and P15 from Pathogen + anc mbiota group and Pathogen only group - using Kruskal-Wallis rank sum test df=3, *P*=0.4689).

**S9c**


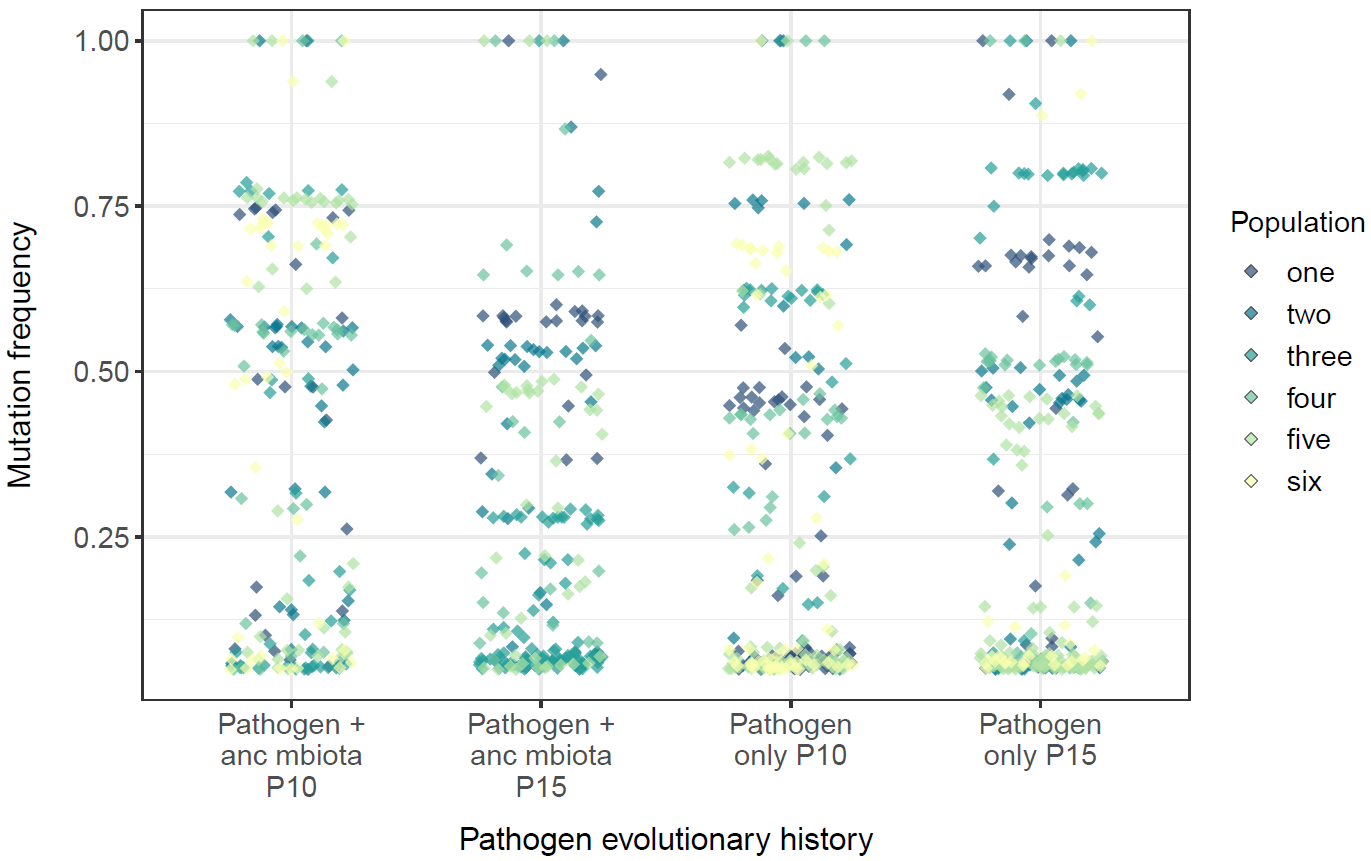


**S9c)** Mutation frequency in each replicate of passages 10 and 15 for each treatment. The frequency of mutations ranged from 5-100%.


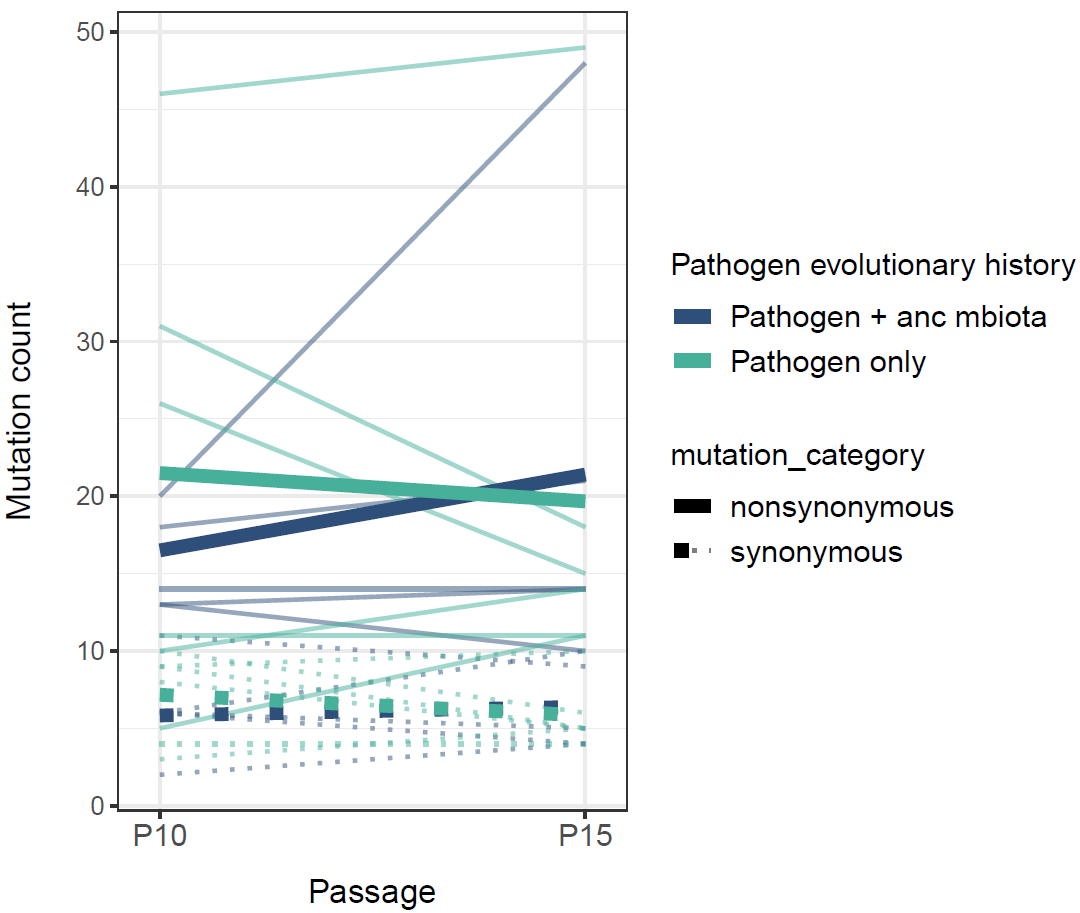


**S9d**

**S9d)** Nonsynonymous and synonymous mutation counts across passages 10 and 15 for each *in vivo* evolved treatment. In both treatments, there were generally more nonsynonymous than synonymous SNPs at each time point.


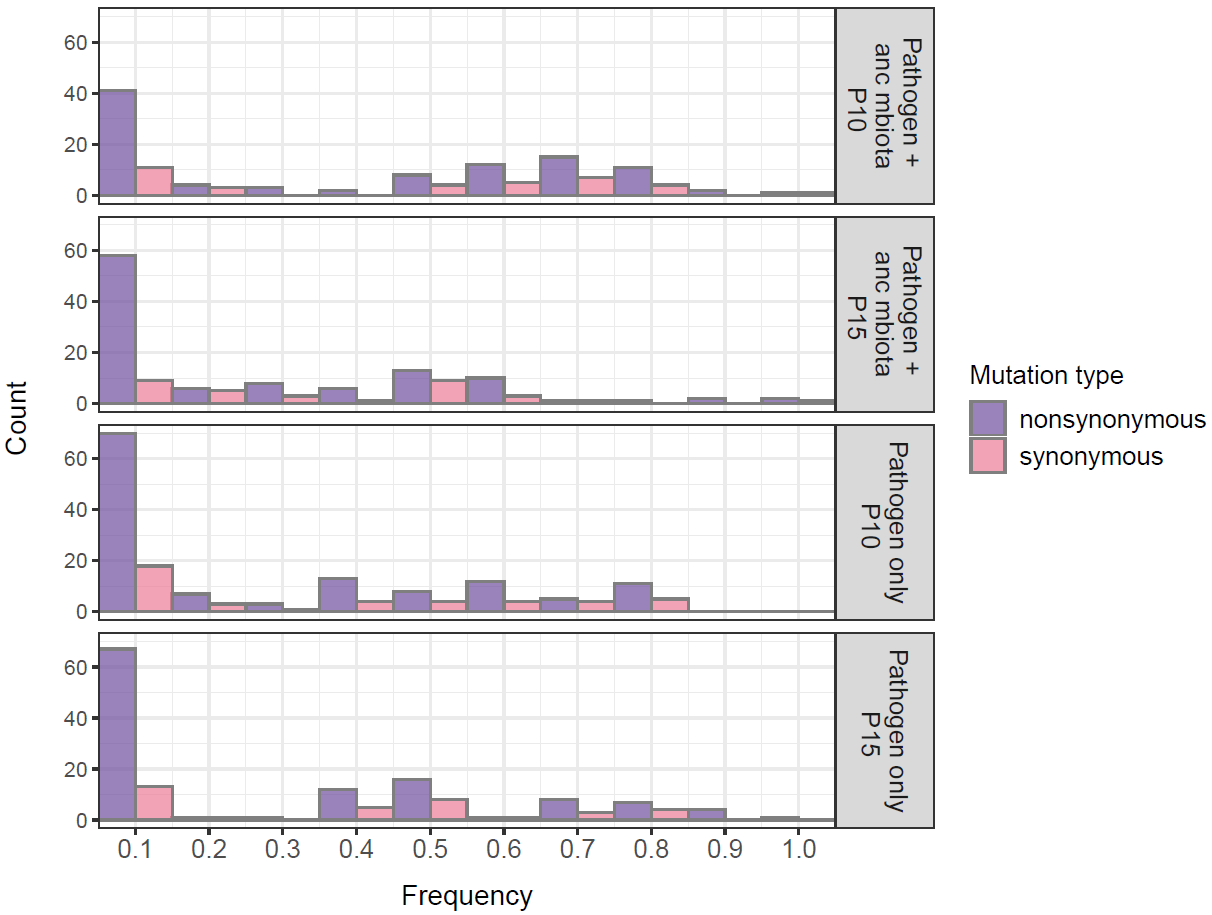


**S9e**

**S9e)** Histogram of nonsynonymous and synonymous mutations for passages 10 and 15 of both treatments. Each tick mark on the x-axis indicates the higher value of that bin. E.g., the tick mark at 0.2 is showing how many non/syn mutations are found between 0.1 and 0.2 frequency.

**S9f**


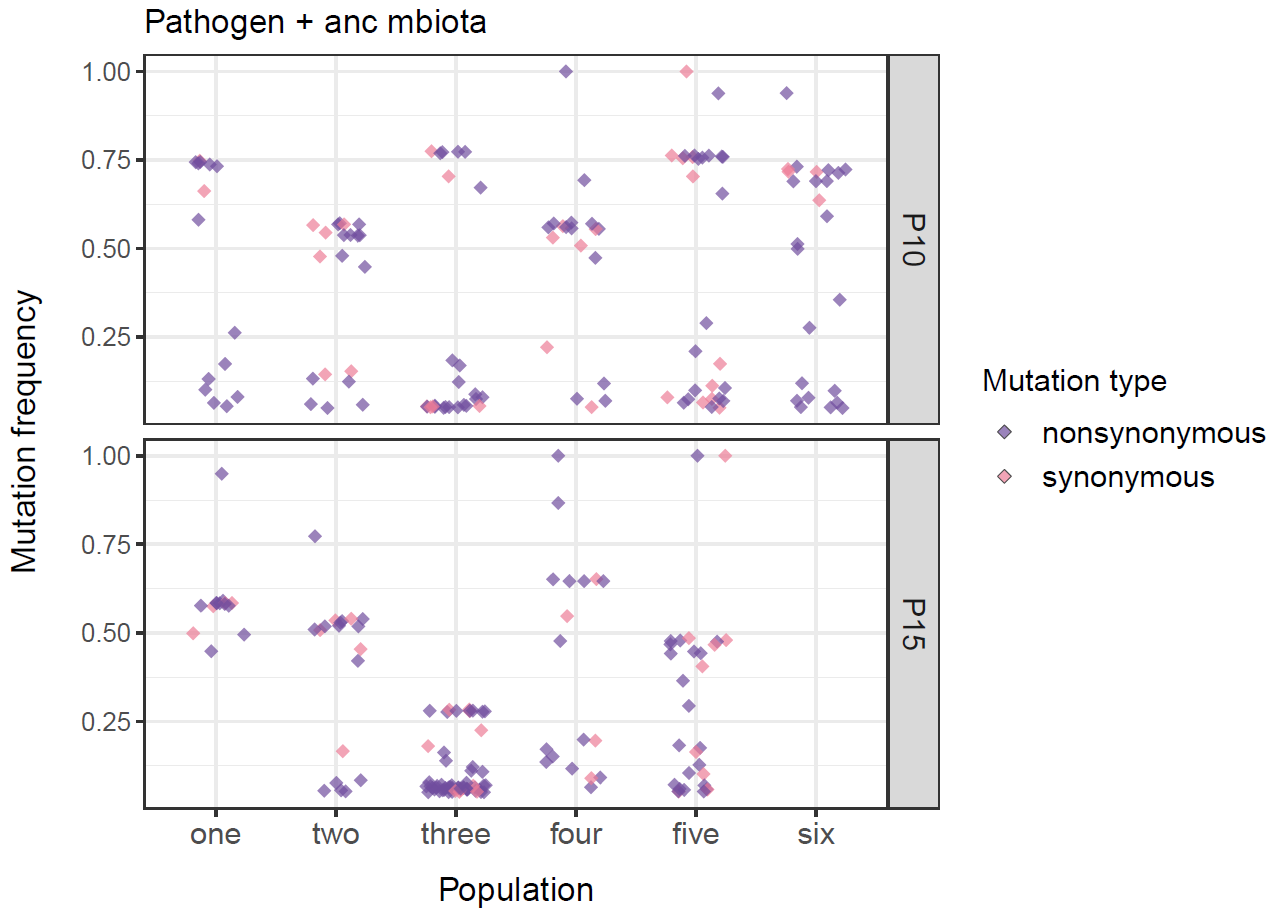


**S9f)** Frequencies of nonsynonymous and synonymous mutations at passages 10 and 15 for the ‘Pathogen + anc mbiota’ treatment. Across populations, there was variation in terms of the frequency of nonsynonymous vs. synonymous SNPs. For example, most mutations in pathogen only P15 population six were at < 25%, whereas populations one and three of the same treatment and time point had many mutations at > 50%.

**S9g**


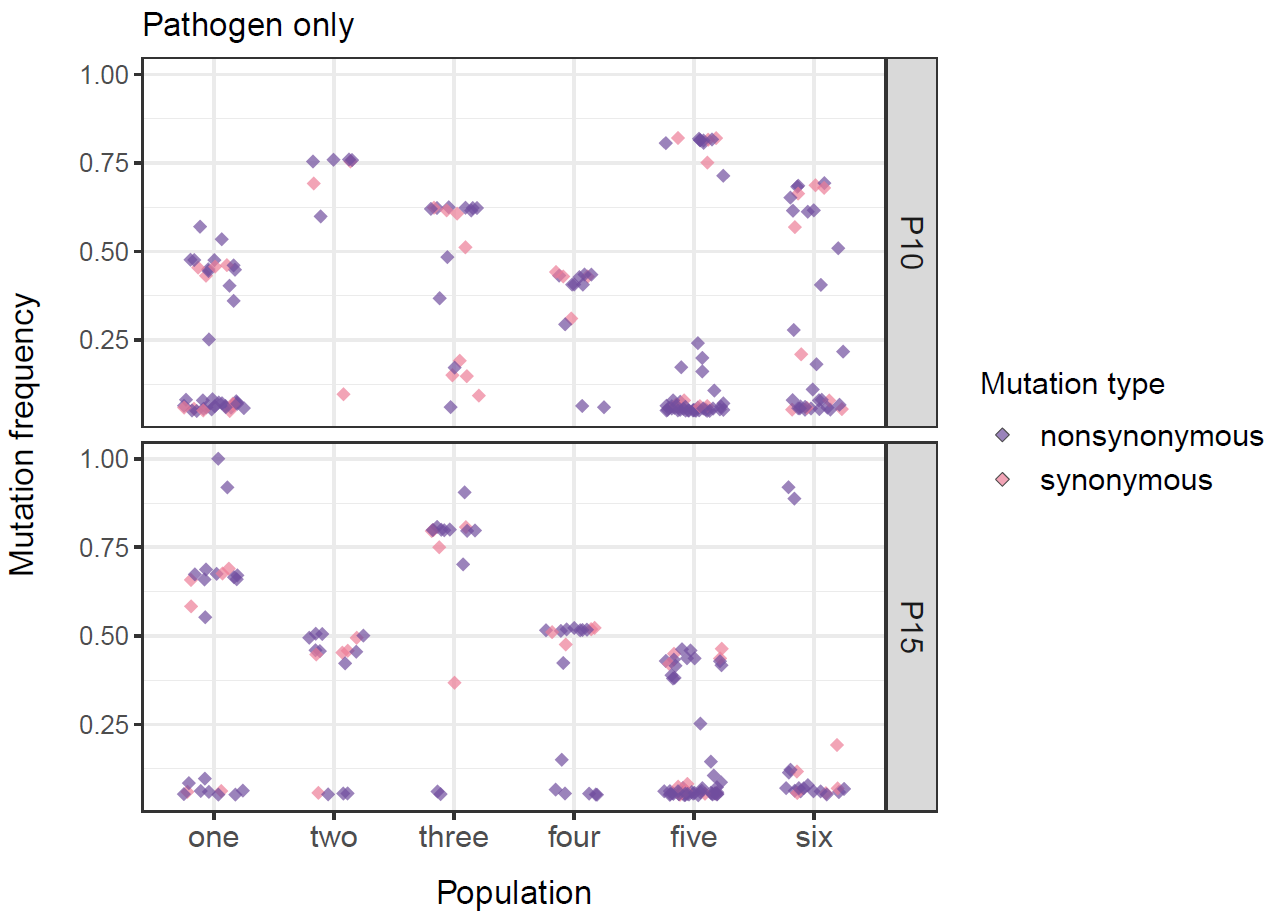


**S9g)** Frequencies of nonsynonymous and synonymous mutations at passages 10 and 15 for the ‘Pathogen only’ treatment. Across populations, there was variation in terms of the frequency of nonsynonymous vs. synonymous SNPs. For example, most mutations in pathogen only P15 population six were at < 25%, whereas populations one and three of the same treatment and time point had many mutations at > 50%.

**S9h**


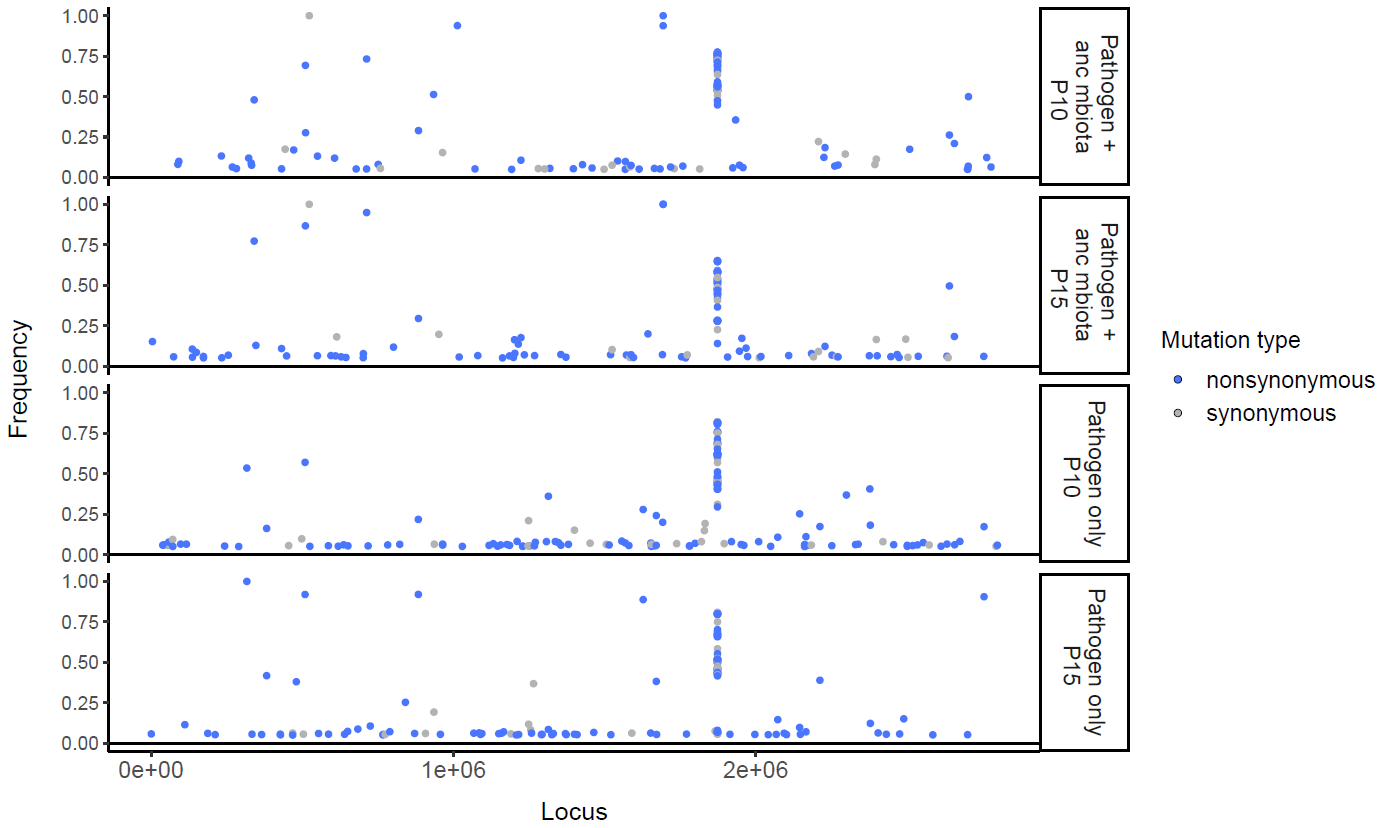


**S9h)** Distribution of nonsynonymous and synonymous mutations across pathogen genome, per treatment for passages 10 and 15.

**Supplementary table 1.** Genes under selection at generation 15 in evolved pathogen lineages, as determined by presence of SNPs at a frequency of >=25% in the population. Populations consisted of 40 clones. Genes with links to biofilm formation are highlighted in bold. All SNPs were unique to one replicate population.

| **SNP position** | **Mutation** | **Gene** | **Broad functional category** | **Specific function** | **Evolution experiment group** | **Replicate population** | **SNP frequency in population** |
| --- | --- | --- | --- | --- | --- | --- | --- |
| 72108 | G-A | SAS_RS00315 ← / → SAS_RS00320 | Metabolism | Energy metabolism | Path + anc mbiota | 2 | 34.5 |
| 340926 | T-A | SAS_RS01520 ← | Metabolism | Carbohydrate metabolism | Path + anc mbiota | 2 | 77.3 |
| 397613 | C-T | SAS_RS01820 → / ← SAS_RS01825 | Metabolism | Stress response | Path + anc mbiota | 2 | 87.0 |
| 510405 | G-A | spoVG → | Metabolism | Cell division | Path + anc mbiota | 4 | 86.7 |
| 523155 | T-C | tilS → | Metabolism | Protein synthesis | Path + anc mbiota | 5 | 100.0 |
| 616348 | A-G | SAS_RS02870 → / → SAS_RS02875 | Metabolism | Fatty acid metabolism | Path + anc mbiota | 3 | 27.0 |
| 712911 | G-T | **SAS_RS03390 →** | Adherence | Adhesion | Path + anc mbiota | 1 | 94.9 |
| 884393 | C-T | **dltB →** | Metabolism | Cell wall biosynthesis | Path + anc mbiota | 5 | 29.4 |
| 972973 | C-T | SAS_RS04700 → / → SAS_RS04705 | Metabolism | DNA metabolism | Path + anc mbiota | 2 | 72.6 |
| 1114130 | G-A | SAS_RS05505 ← / ← SAS_RS05510 | Metabolism | Metal ion transport | Path + anc mbiota | 3 | 27.3 |
| 1693728 | C-G | **SAS_RS08335 ←** | Metabolism | DNA metabolism | Path + anc mbiota | 4 | 100.0 |
| 1693770 | G-T | **SAS_RS08335 ←** | Metabolism | DNA metabolism | Path + anc mbiota | 5 | 100.0 |
| 1757780 | T-A | accD ← / ← SAS_RS08650 | Metabolism | Fatty acid metabolism | Path + anc mbiota | 5 | 29.9 |
| 2640523 | G-A | SAS_RS14945 → | Metabolism | Hypothetical protein | Path + anc mbiota | 1 | 49.5 |
| 316725 | T-A | SAS_RS01385 → | Virulence | Immune evasion | Pathogen only | 1 | 100.0 |
| 382255 | G-T | SAS_RS01725 ← | Metabolism | Protein synthesis | Pathogen only | 5 | 41.7 |
| 480287 | C-T | **SAS_RS02230 →** | Metabolism | Protein synthesis | Pathogen only | 5 | 38.0 |
| 509174 | G-T | purR → | Metabolism | DNA metabolism | Pathogen only | 1 | 91.9 |
| 674451 | G-A | SAS_RS03200 → / ← SAS_RS03205 | Metabolism | Metal ion transport | Pathogen only | 1 | 44.5 |
| 841226 | A-C | **clfA →** | Adherence | Adhesion | Pathogen only | 5 | 25.3 |
| 883866 | G-T | dltB → | Metabolism | Cell wall biosynthesis | Pathogen only | 6 | 92.0 |
| 1164888 | C-A | SAS_RS05775 ← / → SAS_RS05780 | Virulence | Immune evasion | Pathogen only | 5 | 35.9 |
| 1264990 | C-T | dprA → | Metabolism | DNA metabolism | Pathogen only | 3 | 36.8 |
| 1627590 | G-C | recO ← | Metabolism | DNA metabolism | Pathogen only | 6 | 88.7 |
| 1670968 | C-T | SAS_RS08230 ← | Metabolism | DNA metabolism | Pathogen only | 5 | 38.2 |
| 1693911 | T-G | **SAS_RS08335 ← / ← recJ** | Metabolism | DNA metabolism | Pathogen only | 4 | 100.0 |
| 2212293 | G-C | SAS_RS11165 → | Metabolism | Carbohydrate metabolism | Pathogen only | 5 | 38.9 |
| 2755023 | G-A | **icaC →** | Adherence | Adhesion | Pathogen only | 3 | 90.5 |

**Supplementary table 2.** Filtered breseq results. All mutations included are at a frequency of 0.05 to 1.


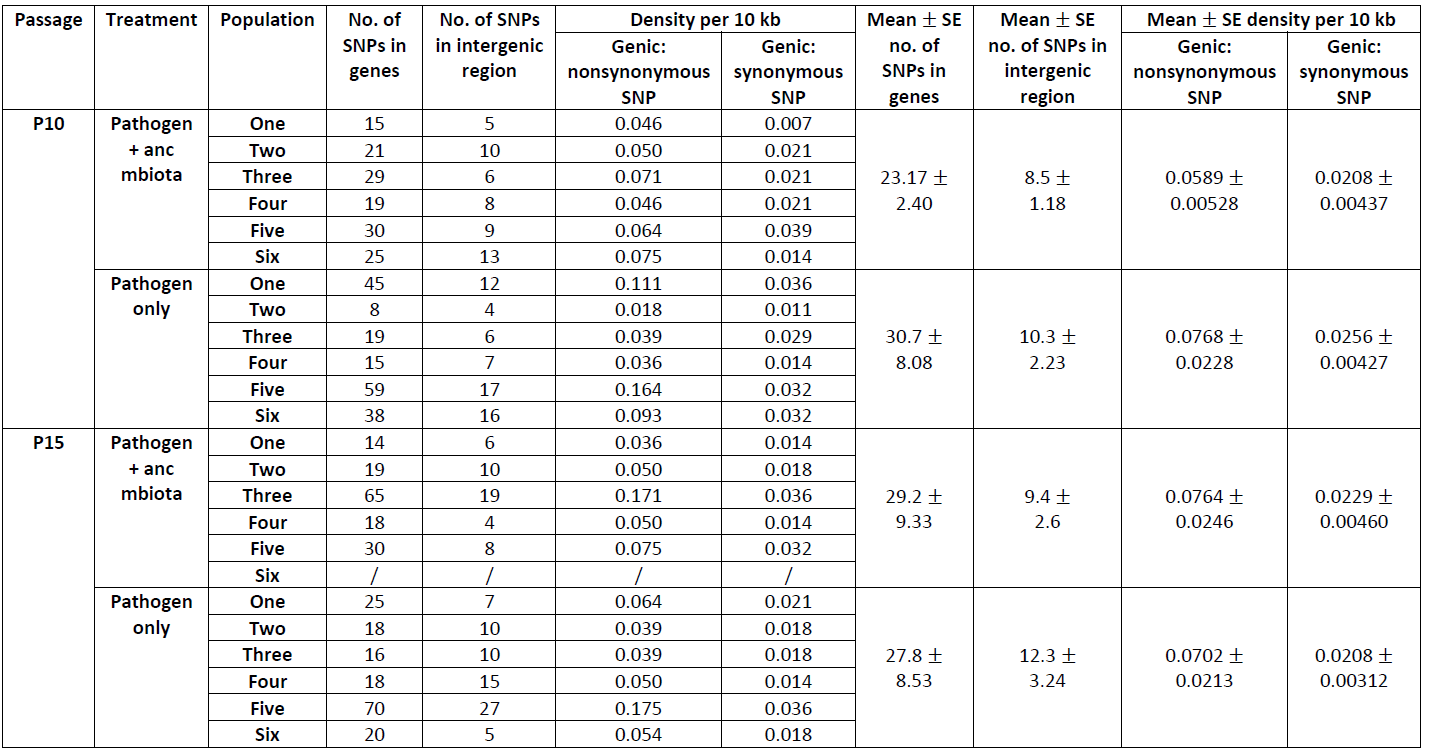

Supplement: 25-4-8_Supplementary_material_and_figures_wraf071 [file 25-4-8_supplementary_material_and_figures_wraf071.docx]
